# Causality Analysis and Cell Network Modeling of Spatial Calcium Signaling Patterns in Liver Lobules

**DOI:** 10.3389/fphys.2018.01377

**Published:** 2018-10-04

**Authors:** Aalap Verma, Anil Noronha Antony, Babatunde A. Ogunnaike, Jan B. Hoek, Rajanikanth Vadigepalli

**Affiliations:** ^1^Department of Biomedical Engineering, University of Delaware, Newark, DE, United States; ^2^Department of Pathology, Anatomy and Cell Biology, Daniel Baugh Institute for Functional Genomics and Computational Biology, Thomas Jefferson University, Philadelphia, PA, United States; ^3^Department of Chemical and Biomolecular Engineering, University of Delaware, Newark, DE, United States

**Keywords:** calcium dynamics, liver lobule, causal network analysis, computational modeling, spatial calcium patterns, cell-cell interactions

## Abstract

Dynamics as well as localization of Ca^2+^ transients plays a vital role in liver function under homeostatic conditions, repair, and disease. In response to circulating hormonal stimuli, hepatocytes exhibit intracellular Ca^2+^ responses that propagate through liver lobules in a wave-like fashion. Although intracellular processes that control cell autonomous Ca^2+^ spiking behavior have been studied extensively, the intra- and inter-cellular signaling factors that regulate lobular scale spatial patterns and wave-like propagation of Ca^2+^ remain to be determined. To address this need, we acquired images of cytosolic Ca^2+^ transients in 1300 hepatocytes situated across several mouse liver lobules over a period of 1600 s. We analyzed this time series data using correlation network analysis, causal network analysis, and computational modeling, to characterize the spatial distribution of heterogeneity in intracellular Ca^2+^ signaling components as well as intercellular interactions that control lobular scale Ca^2+^ waves. Our causal network analysis revealed that hepatocytes are causally linked to multiple other co-localized hepatocytes, but these influences are not necessarily aligned uni-directionally along the sinusoids. Our computational model-based analysis showed that spatial gradients of intracellular Ca^2+^ signaling components as well as intercellular molecular exchange are required for lobular scale propagation of Ca^2+^ waves. Additionally, our analysis suggested that causal influences of hepatocytes on Ca^2+^ responses of multiple neighbors lead to robustness of Ca^2+^ wave propagation through liver lobules.

## Introduction

The liver performs a wide variety of physiological functions, including the regulation of intermediary metabolism, lipid synthesis, bile production, and xenobiotic detoxification. Normal liver function requires both tight regulation of intracellular processes and intercellular coordination. Free Ca^2+^ in the intracellular domain participates in the regulation of such hepatocyte functions as glucose metabolism, bile secretion, proliferation, and apoptosis (Exton, 1987; McConkey and Orrenius, 1997; Canaff et al., 2001). Regulation of cytosolic Ca^2+^ is particularly important in hepatocytes, the cells responsible for the bulk of metabolic and detoxification activities in the liver. Consequently, disruption of Ca^2+^ dynamics can potentially lead to pathological conditions, such as cholestasis (Kruglov et al., 2011).

Structurally, cells in the liver are arranged in lobules, the functional units of the liver conceptualized as having roughly hexagonal cross sections delineated by the portal triad and the central veins. Circulating blood enters lobules through the portal vein and hepatic artery residing in the periportal region, and is drained into the central vein after passing through sinusoids. Hepatocytes are polarized cells arranged alongside sinusoids, with their basolateral membranes in contact with the systemic blood flow. Upon contact with Ca^2+^ mobilizing agents in the blood stream, such as ATP, hormones, or growth factors, spikes in cytosolic Ca^2+^ concentration are observed within the intracellular domains of hepatocytes (Woods et al., 1986, 1987; Serradeil-Le Gal et al., 1991). Binding of extracellular stimuli, such as hormones to receptors on the basolateral hepatocyte membranes, elicits an intracellular signaling cascade involving phospholipase C (PLC) activation, inositol triphosphate (IP_3_) synthesis, IP_3_ receptor (IP_3_R) activation in the endoplasmic reticulum (ER) membrane, leading to a rapid efflux of Ca^2+^ from the ER into the cytosol. Once in the cytosol, Ca^2+^ can be sequestered by mitochondria, released into the extracellular region, or pumped back into the ER, thus reducing the cytosolic Ca^2+^ levels, yielding a Ca^2+^ spike. Intracellular Ca^2+^ spiking has been reported to arise primarily due to fast activation and slow inhibition of IP_3_R by cytosolic Ca^2+^ operating in conjunction with active pumping of cytosolic Ca^2+^ into the ER by SERCA pumps (Atri et al., 1993; Keizer and De Young, 1993). In the intact rat liver, sustained hormone stimulation typically leads to Ca^2+^ spike trains in hepatocytes with inter-spike intervals dependent upon stimulus strength as well as intracellular signaling capacity (Robb-Gaspers and Thomas, 1995).

Heterogeneity in the expression and intracellular distribution of the Ca^2+^ signaling components as well as variability in the extracellular regulatory factors can lead to differences in characteristic Ca^2+^ spiking frequencies of individual hepatocytes. Variations in Ca^2+^ spike frequencies have been shown to lead to differential downstream gene expression and protein regulation (Dolmetsch et al., 1998; Zhu et al., 2008; Smedler and Uhlén, 2014). A coherent lobular scale response to extracellular stimuli requires that the Ca^2+^ signals in hepatocytes across the lobule be coordinated. Gap junctions are hypothesized to play a role in coordinating this response, leading to synchronization of cytosolic Ca^2+^ spikes across the liver lobule in response to G-protein coupled receptor agonists (Robb-Gaspers and Thomas, 1995; Tordjmann et al., 1997). Cytosolic Ca^2+^ and IP_3_ from a hepatocyte can migrate to neighboring hepatocytes, likely responsible for inducing synchronization of Ca^2+^ spikes across the liver lobule (Sáez et al., 1989). Another mechanism of long-range coordination may involve the release of paracrine signals, such as ATP, into the extracellular milieu, which then elicits Ca^2+^ spiking in the neighboring hepatocytes by purinergic receptor activation (Schlosser et al., 1996). Exchange of molecular signals between cells through gap junctions or paracrine signaling in addition to spatially organized heterogeneity could lead to a coordinated response within a population of cells with regard to downstream processes regulated by Ca^2+^ in response to extracellular stimuli.

In liver lobules, Ca^2+^ signals commonly manifest as traveling waves (Keizer and De Young, 1993; Robb-Gaspers and Thomas, 1995; Thomas et al., 1996). Ca^2+^ waves usually start in cells located in the pericentral (PC) region of the lobule and propagate toward the periportal (PP) region (Nathanson et al., 1995; Robb-Gaspers and Thomas, 1995) upon lobule-wide stimulation by vasopressin or phenylephrine. The direction of Ca^2+^ signal propagation is opposite to the general direction of blood flow, which is from PP to PC. This observation indicates an organized spatial heterogeneity, termed liver zonation, in the Ca^2+^ signaling capacity of cells. Liver zonation has been observed in many other physiological functions in liver lobules (Gebhardt and Mecke, 1983; Jungermann, 1987; Braeuning et al., 2006; Gebhardt and Matz-Soja, 2014).

Dynamics as well as localization of Ca^2+^ transients plays a vital role in liver function under homeostatic conditions, repair, and disease (Rooney et al., 1990; Pusl and Nathanson, 2004; Gaspers and Thomas, 2008; Lagoudakis et al., 2010; Fu et al., 2012; Amaya and Nathanson, 2013; Bartlett et al., 2014; Oliveira et al., 2015). Although a wealth of information exists regarding the intracellular Ca^2+^ dynamics, understanding of lobular scale propagation of Ca^2+^ signal, its quantification as well as a clear understanding of its significance is lacking. Use of computational modeling to decipher processes that control intracellular Ca^2+^ spikes and spatial patterns of Ca^2+^ signal propagation has been a long-standing area of investigation due to the complexity of their origin and propagation. For instance, Schuster et al. (2002) present a detailed discussion of computational models developed for describing Ca^2+^ spiking, as well as propagation of Ca^2+^ waves through co-localized cells. In previous work, we used a computational model to predict that zonation of intracellular signaling components as well as gap junction-mediated IP_3_ exchange between immediate neighbors are required for the propagation of a Ca^2+^ signal through a chain of connected hepatocytes (Verma et al., 2016). A recent study employing single cell RNA sequencing provided evidence in support of our predictions of lobular gradients of intracellular signaling components at the mRNA level (Halpern et al., 2017).

In this study, we combined analysis of experimentally acquired images of cytosolic Ca^2+^ dynamics in mouse liver lobules with dynamic modeling to identify spatial features of lobular scale Ca^2+^ signal propagation and putative causal linkages between adjacent hepatocytes. We imaged cytosolic Ca^2+^ levels in response to a vasopressin stimulus in a 2D optical slice of a perfused intact mouse liver to obtain a data set on Ca^2+^ transients in 1300 hepatocytes residing in different lobules, measured every 4 s over a period of 1600 s. We analyzed correlation as well as causal networks constructed using the acquired high-dimensional time series to characterize the spatial extent and directional alignment of intercellular interactions that lead to Ca^2+^ waves across liver lobules. We incorporated the causal connectivity from network analysis into an ordinary differential equation-based dynamic model of intra-and inter-cellular Ca^2+^ signaling. We utilized the model to evaluate the effect of spatial heterogeneity in the intra- and inter-cellular signaling components on spatial patterns of cytosolic Ca^2+^ signals. Our dynamic model-based analysis predicted the spatial distribution of signaling components that yield lobular scale Ca^2+^ patterns that are consistent with the experimentally observed Ca^2+^ wave propagation.

## Methods

### Calcium imaging in isolated perfused mouse livers

All animal procedures used in this study were handled in accordance with mandated standards of humane care and were approved by the Thomas Jefferson University Institutional Animal Care and Use Committee. Confocal imaging of intact perfused livers was performed as previously described (Robb-Gaspers and Thomas, 1995; Bartlett et al., 2017). Briefly, livers from 8–12 weeks old male C57 BL/6 mice were perfused via the hepatic portal vein with a HEPES-buffered balance salt solution (121 mM NaCl, 25 mM HEPES, 5 mM NaHCO3, 4.7 mM KCl, 1.2 mM KH_2_PO_4_, 1.2 mM MgSO_4_, 1.3 mM CaCl_2_, 5.5 mM glucose, 0.5 mM glutamine, 3 mM lactate, 0.3 mM pyruvate, 0.2 mM bromosulfophthalein (BSP), 0.1% BSA, pH 7.4) equilibrated with 100% O_2_ at 30°C. A Ca^2+^-sensitive indicator, fluo-8 AM (5 μM) was loaded into the hepatocytes *in vivo* by recirculating the perfusion buffer supplemented with fluo-8 AM plus 0.02% Pluronic F-127 and 2% BSA for 40–50 min. Confocal images were acquired with an EC Plan-Neofluar 10x/0.30 M27 objective using a Zeiss LSM510MP confocal microscope. Fluo-8 images (488 nm excitation, 520–600 nm emission) were captured every 4 s. Periportal and pericentral zones were identified by differential dye loading and perfusion of fluorescein-conjugated BSA.

### Image segmentation and cytosolic calcium time trace extraction

Hepatocytes in the acquired images were segmented manually. Intensities of all pixels lying within segmented hepatocyte boundaries were added for every time slice to obtain a 400-time point cytosolic Ca^2+^ time series for all the segmented hepatocytes.

### Pre-processing cytosolic Ca^2+^ time series data

The following operations were performed on the cytosolic Ca^2+^ trace of every hepatocyte (see Supplementary Figure S1 for details):

#### Baseline correction and rescaling

The cytosolic Ca^2+^ series for each hepatocyte was detrended using an implementation of the rolling ball baseline correction algorithm contained in the *baseline* package (version 1.2) in R platform for statistical analysis (version 3.2.3; R Core Team, 2015) to remove low frequency components and correct for dye photobleaching during the experiment.

#### Low pass filtering and rescaling

High frequency components in each baseline-corrected Ca^2+^ time series were removed using the *smooth.fft* function from the *itsmr* package (version 1.5) in R (version 3.2.3). This function removes frequencies corresponding to the highest *n*th percentile from the power spectrum of a given time series. The signal in the lowest 27.5 percentile of the frequency range in every time series was retained for subsequent analysis. The amplitudes of cytosolic Ca^2+^-dependent fluorescence signal intensity in the resultant time series were then rescaled to between 0 and 1.

### Network analysis

#### Undirected correlation network construction and analysis

Undirected correlation networks were constructed using pairwise Spearman rank correlation coefficient estimates of baseline corrected, low pass filtered, and rescaled time series data. Edges corresponding to correlation values < 0.75 or those that were between hepatocytes lying at a distance > 100 μm were discarded. The resultant networks were analyzed for isolated clusters, their sizes and node degrees. Analysis of the correlation network was performed using the *igraph* (version 1.0.1) package in R (version 3.2.3).

#### Transfer entropy (TE) based causal network construction and analysis

Transfer entropy (TE) is a measure of the directed influence between two random processes. TE from a process X to another process Y is defined as the amount of reduction in uncertainty of future values of Y by knowing the past values of X, given past values of Y. In the present study, pair-wise TE between Ca^2+^ responses of hepatocytes was estimated based on Shannon's conditional entropy, as follows:

(1) $$\begin{array}{rcl} h_{1} & = & -\sum_{y_{t+1},Y_{t}^{n},X_{t}^{m}}p\left(y_{t+1},Y_{t}^{n},X_{t}^{m}\right).\log p\left(y_{t+1}|Y_{t}^{n},X_{t}^{m}\right) \end{array}$$

(2) $$\begin{array}{rcl} h_{2} & = & -\sum_{y_{t+1},Y_{t}^{n},X_{t}^{m}}p\left(y_{t+1},Y_{t}^{n},X_{t}^{m}\right).\log p\left(y_{t+1}|Y_{t}^{n}\right) \end{array}$$

(3) $$\begin{array}{rcl} TE_{X\to Y} & = & h_{2}-h_{1} \end{array}$$

where *x*_*t*_ and *y*_*t*_ represent the cytosolic Ca^2+^ levels in hepatocytes X and Y, respectively, at time *t*; $X_{t}^{m}=\left[x_{t},x_{t-1},\ldots ,x_{t-m+1}\right]$ and $Y_{t}^{n}=\left[y_{t},y_{t-1},\ldots ,y_{t-n+1}\right]$ are past m and n values of cytosolic Ca^2+^ in respective hepatocytes X and Y; $p\left(y_{t+1}|Y_{t}^{n},X_{t}^{m}\right)$ is the probability of the occurrence of *y*_*t*+1_ given $X_{t}^{m}$ and $Y_{t}^{n}$, and $p\left(y_{t+1},Y_{t}^{n},X_{t}^{m}\right)$ is the joint probability of occurrence of *y*_*t*+1_, $X_{t}^{m}$, and $Y_{t}^{n}$. TE therefore represents the decrease in Shannon's entropy when past values of X *and* Y are used to predict the current value of Y compared to past values of Y alone. Information transfer is considered as occurring from X to Y if *TE*_*X*→*Y*_> 0 (see Schreiber, 2000 for more details). In this work, m and n were taken to be 1 based on cross correlation measures (Figure S2). Additionally, a theoretical estimate of IP_3_ diffusion time between two hepatocytes with diameters of ~ 25 μm is 1.1 s based on IP_3_ diffusion constant values in xenopus oocyte cytosolic extracts (Allbritton et al., 1992). We therefore limited our TE analysis to a history value of 1 (4 s lag), with information flow interpreted as IP_3_ exchange between neighboring hepatocytes. It must be noted that the diffusion time of IP_3_ between hepatocytes *in vivo* might be increased due to molecular charge, gap junction channel properties and cell-intrinsic buffering. However, to our knowledge, no data exists regarding effective diffusivity of IP_3_ in mouse hepatocytes *in vivo*.

Directed causal networks between hepatocytes based on TE were constructed using quantized Ca^2+^ time series of all hepatocytes. Pre-processed Ca^2+^ time series for each hepatocyte was quantized into high (= 1) or low (= 0) cytosolic Ca^2+^ at each time point using the 85th percentile of the cell-intrinsic intensity distribution as considered the threshold. As an additional filter to minimize the effect of noise, all high cytosolic Ca^2+^ values in the time series were changed to zero unless the value at an immediately preceding or following time point was also high, i.e., cytosolic Ca^2+^ intensity was sustained above the 85th percentile within the cell for at least 8 s.

In the absence of a good value of TE to infer cell-to-cell influence, hepatocyte-specific significance testing was employed to identify influence edges and construct TE-based causal networks. For every hepatocyte, pairwise TE values from all other hepatocytes were estimated to obtain an empirical distribution. If the TE value to the given hepatocyte from another adjacent hepatocyte was greater than the 95th percentile of its empirical TE distribution, a positive causal influence was considered from the neighbor to the hepatocyte of interest. The similarity in TE networks identified by our method based on binarized data and a continuous TE estimation method implemented in JIDT (Lizier, 2014) can be found in Figure S11. Additionally, we chose a cell-specific TE threshold instead of a global threshold to avoid inclusion of false positives (Figure S12).

### Computational modeling of intra- and inter-cellular Ca^2+^ signaling

We started with a receptor oriented, ordinary differential equation (ODE)-based model of Ca^2+^ signal propagation in a cord of hepatocytes detailed in Verma et al. (2016). Here, we consider the complex spatial features of a liver lobule by allowing the hepatocytes to be connected with more than two other hepatocytes, as was the case in the original model of Verma et al. (2016). In the present computational model, the state of every cell “i” and its interaction with a set of adjacent cells represented by the index “j” is defined by the following system of ODEs:

(4) $$\begin{array}{rcl} \frac{dr_{i}}{dt} & = & k_{r_{i}}\left(1-r_{i}\right)-k_{d}r_{i}-k_{Hr}H.r_{i} \end{array}$$

$$\begin{array}{rcl} \frac{dIP3_{i}}{dt} & = & \left(\frac{k_{IP3_{i}}.H.r_{i}}{k_{cat}+r_{i}}\right)\left(1-\frac{k_{3}}{CaI_{i}+k_{3}}\right)-D\frac{IP3_{i}}{2} \end{array}$$

(5) $$\begin{array}{rc} - & \underset{j\in adj_{i}}{\sum }G_{ij}\left(IP3_{i}-IP3_{j}\right) \end{array}$$

$$\begin{array}{rcl} \frac{dCaI_{i}}{dt} & = & \left(1-g_{i}\right)\left(\frac{A{\left(\frac{IP3_{i}}{2}\right)}^{4}}{{\left(k_{1}+\frac{IP3_{i}}{2}\right)}^{4}}+L\right)\left(CaT_{i}-CaI_{i}\right) \end{array}$$

(6) $$\begin{array}{rc} - & \frac{B.CaI_{i}^{2}}{k_{2}^{2}+CaI_{i}^{2}} \end{array}$$

(7) $$\begin{array}{rcl} \frac{dg_{i}}{dt} & = & E.CaI_{i}^{4}\left(1-g_{i}\right)-F \end{array}$$

(8) $$\begin{array}{rcl} \frac{dCaT_{i}}{dt} & = & -\frac{dCaI_{i}}{dt} \end{array}$$

Surface receptor activity (*r*) including non-specific binding was modeled as shown in Equation (4), where H represents the hormone level—a model parameter. The total number of receptors for each hepatocyte was assumed to be constant. Intracellular IP_3_ concentration (*IP3*) balance was modeled using saturation kinetics for synthesis influenced by hormone binding to the receptor and cytosolic Ca^2+^ and mass action kinetics for degradation (Equation 5). IP_3_ exchange between adjacent hepatocytes was modeled as a mass transfer term assuming fast kinetics, with G_ij_ being the mass transfer coefficient. Increase in cytosolic Ca^2+^ (*CaI*) in the model was regulated IP_3_R (*g*) activation, cytosolic IP_3_ levels, store Ca^2+^ content (*CaT*), and a constant leakage from the ER (*L*), whereas decrease in cytosolic Ca^2+^ caused by SERCA pump activity was modeled as a Hill function (Equation 6). IP_3_R activation (*g*) in the model was regulated by cytosolic Ca^2+^, whereas a constitutive rate of IP_3_R was considered (Equation 7). The model assumed constant total intracellular Ca^2+^ for all hepatocytes. Additional details of model development can be found in Verma et al. (2016). See Tables 1, 2 for parameter descriptions, their nominal ranges and initial values for model species. All simulations in this study were performed using Matlab (version 8.1.0.604 (R2013a) Mathworks, Natick, MA).

**Table 1:** List of species and their initial values in the computational model.

**Symbol**	**Value**	**Quantity**
*CaI_*i*_*	0.2 μM	Cytosolic Ca^2+^ concentration
*CaT_*i*_*	500 μM	Total Ca^2+^ concentration
*g_*i*_*	0.25	Ratio of free to total IP_3_R
*IP3_*i*_*	0.1 μM	IP_3_ concentration
*r_*i*_*	0.5	Ratio of free to total agonist receptors in cell i

**Table 2:** List of nominal parameter values/ranges for the computational model.

**Symbol**	**Value**	**Quantity**
*A*	0.20 μM/s	Maximal rate of Ca^2+^ release from ER store
*B*	0.082 μM/s	Maximal rate of cytosolic Ca^2+^ pump to ER
*D*	1.6/s	IP_3_ degradation rate
*E*	1/(μM)^4^-s	IP_3_R deactivation rate
*F*	0.01/s	IP_3_R activation rate
*G*	0–5/s	Mass transfer coefficient between cells i and j
*H*	1.8 × 10^−10^ M	Hormone concentration
*k_1_*	0.5 μM	IP_3_ concentration for half maximal rate of catalysis of store Ca^2+^ release
*k_2_*	0.15 μM	Cytosolic Ca^2+^ concentration for half maximal pump rate
*k_3_*	1 μM	Cytosolic Ca^2+^ concentration for half maximal rate of IP3 production catalysis
*k_*cat*_*	0.45	Bound receptor ratio for half-maximal IP_3_ production rate
*k_*d*_*	0.34/s	Hormone independent agonist receptor binding rate
*k_*Hr*_*	1 /10^−10^ M-s	Hormone-receptor binding rate
*k*_*IP*3_*i*__	0.7 × 10^4^ – 0.9 × 10^4^ μM /s	Saturation IP_3_ synthesis rate
*k*_*r*_*i*__	1–2/s	Agonist receptor recycling rate
*L*	0.00015 μM /s	Ca^2+^ leakage flux from store to cytosol

To identify the effects of non-uniformity of gap junction conductivity between adjacent hepatocytes in our simulations, G_ij_ values were sampled as follows: a uniform random number r_1_ ϵ [0, 1] was drawn. If r_1_ exceeded a threshold value p_th_ (two cases considered: p_th_ = 0.2 or 0.5), a G_ij_ was sampled ϵ [0.5, 0.9]. Otherwise G_ij_ = 0. p_th_ = 0.2 or 0.5 correspond to cases where 20% or 50% G_ij_ values are likely to be 0 respectively.

### Model reproducibility and comparison of alternatives

Simulation results presented in the current work were reproduced independently using the parameter values and hepatocyte adjacency information provided as Supplementary Material with this manuscript. While the original model was implemented in Matlab as a sequentially updating model species according to their specific rate equations, the rate equations in the reproduced model were implemented as a matrix. The Matlab code for the two independent implementations is provided in the Supplementary Material). The simulation results of the two model implementations were in agreement (see Figure S3 for details).

We also considered an alternative modeling scheme, in which the store Ca^2+^ content of each hepatocyte is considered to be a constant (= 500 μM). In this alternative model, Equation (8) is excluded and Equation (6) was changed as follows:

$$\begin{array}{rcl} \frac{dCaI_{i}}{dt} & = & \left(1-g_{i}\right)\left(\frac{A{\left(\frac{IP3_{i}}{2}\right)}^{4}}{{\left(k_{1}+\frac{IP3_{i}}{2}\right)}^{4}}+L\right)\left(500-CaI_{i}\right) \end{array}$$

(6a) $$\begin{array}{rc} - & \frac{B.CaI_{i}^{2}}{k_{2}^{2}+CaI_{i}^{2}} \end{array}$$

## Results

**Figure 1:**
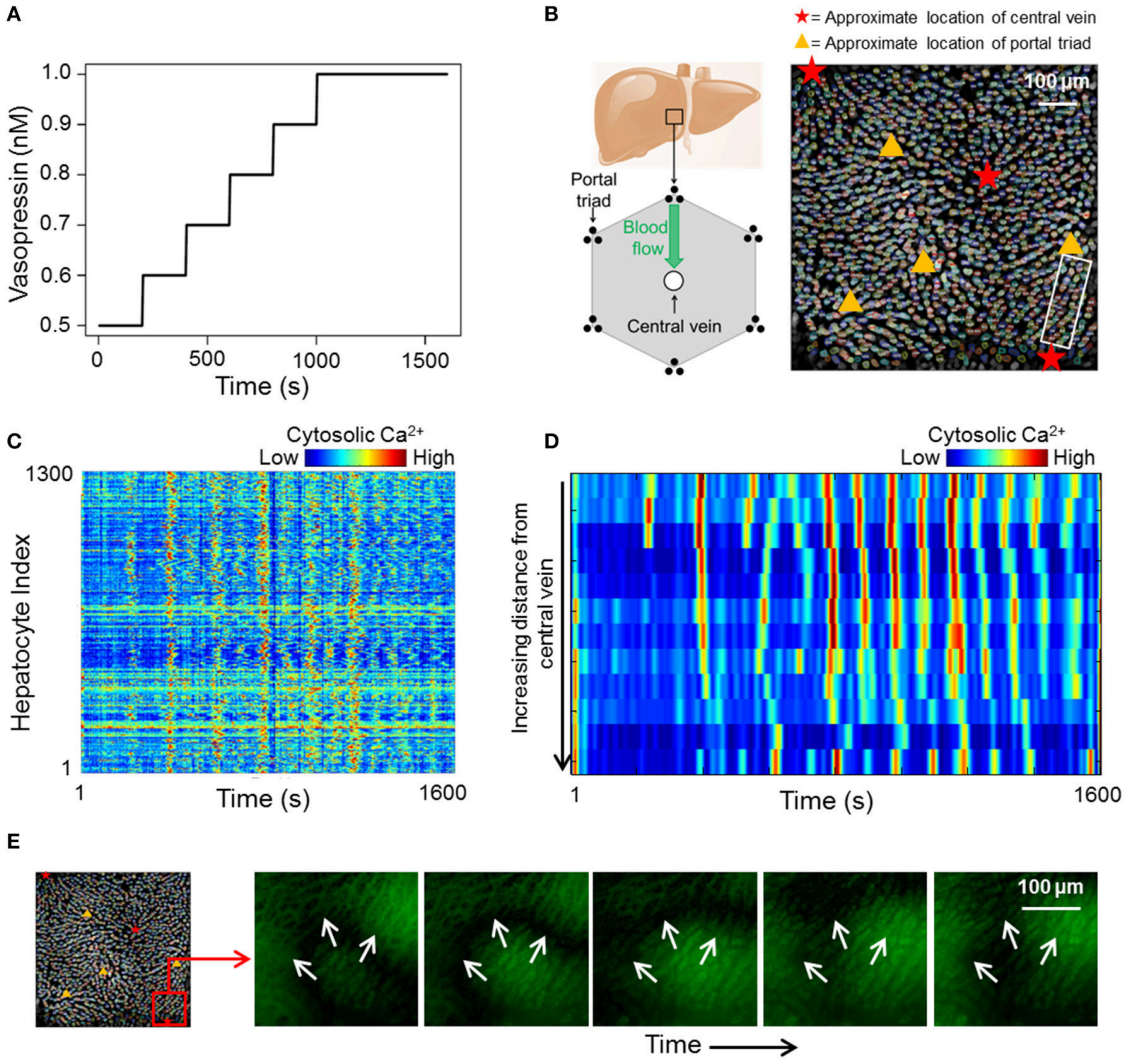
**(A)** vasopressin stimulation profile used during the course of the experiment; **(B)** Left: 2D cross section of an idealized liver lobule with locations of portal triads and central vein. Blood flows from the periportal region toward the pericentral region. Right: Manually segmented hepatocytes with approximate locations of central veins and portal triads in the imaged area. Different colors represent different manually segmented hepatocytes. The hepatocytes are distributed across different liver lobules; **(C)** heat map of cytosolic calcium intensities across all 1,300 segmented hepatocytes over 1,600 s. The data suggests an overall synchronization of calcium spikes across the imaged area; **(D)** Sequential cytosolic Ca^2+^ spiking in 10 hepatocytes residing in the region between a central vein and a portal triad. All hepatocytes are in the region within the white box in **(B)**. Propagating Ca^2+^ waves interspersed with asynchronous spiking can be seen; **(E)** Ca^2+^ wave propagating away from a central vein.

We acquired a dataset consisting of cytosolic Ca^2+^ dynamics in 1300 hepatocytes across different liver lobules over a period of 1600 s (Figure 1). The Ca^2+^ transients within the lobules were induced by a sustained vasopressin stimulus (see section Methods). At low vasopressin stimulus levels (0.1–0.5 nM), hepatocytes in intact mouse livers did not exhibit sustained cytosolic Ca^2+^ spikes (Figure S5). Vasopressin levels to which cells were exposed during the experiment were varied from 0.5 to 1 nM. The stimulus time profile is shown in Figure 1A. We used a step-wise increasing stimulus profile to identify cell-cell interactions that remain unaffected by a change in stimulus strength. Ca^2+^ response profiles for all hepatocytes in our data suggested an overarching synchronized response (Figure 1C). Cytosolic Ca^2+^ spikes as well as Ca^2+^ wave propagation through a lobular section bounded by a central vein and a portal triad are shown in Figures 1D,E, respectively. Hepatocytes generally exhibit asynchronous cytosolic Ca^2+^ spiking behavior superimposed on propagating Ca^2+^ waves.

### Correlation network analysis

**Figure 2:**
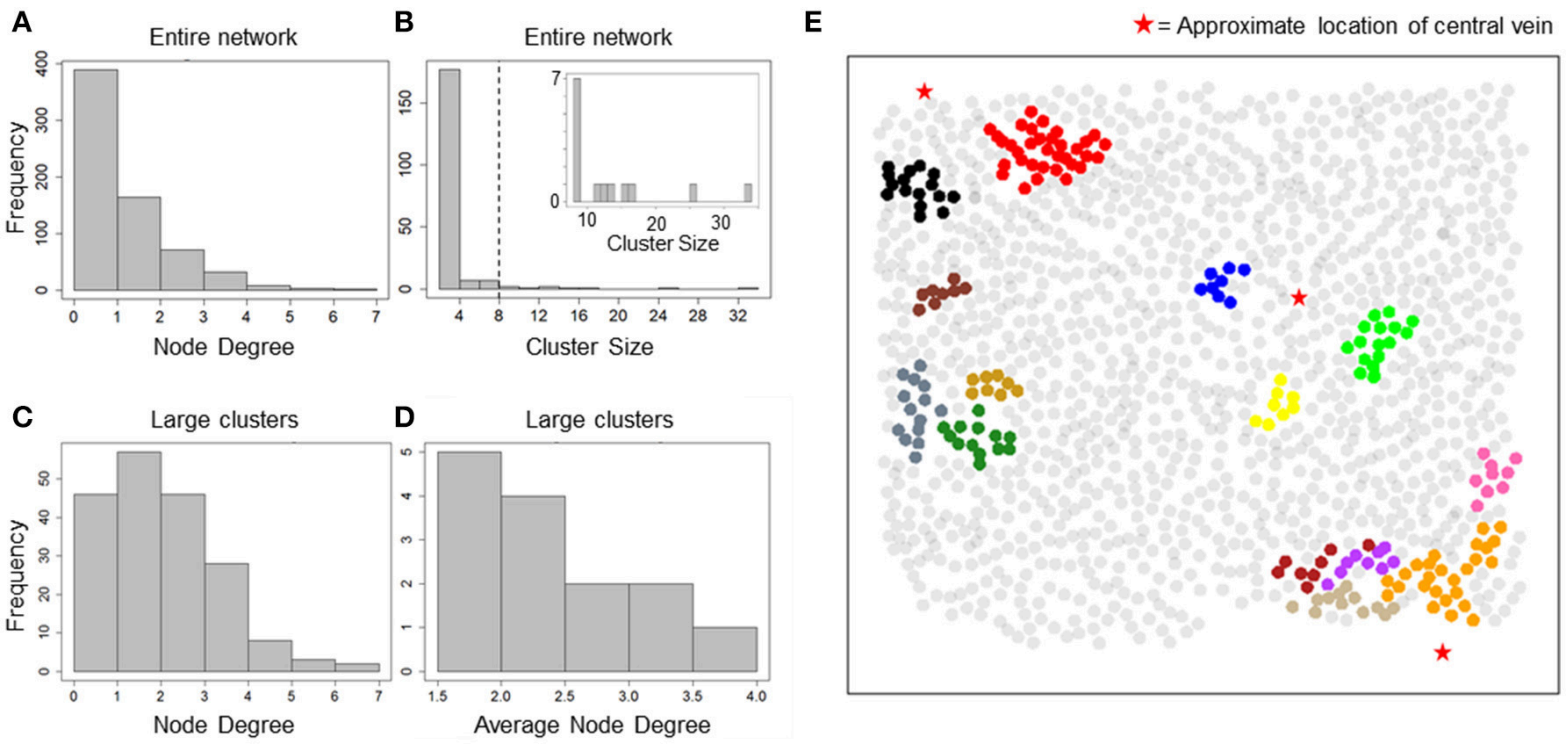
Cluster sizes and degree distribution for correlation-based network. **(A)** Node degree distribution for all nodes in the correlation network. A majority of nodes exhibit a degree < = 2; **(B)** cluster size distribution for all isolated clusters. Most clusters consist of 2 nodes and 1 edge. 14 clusters consisted of > = 8 hepatocytes (inset); **(C)** Node degree distribution for hepatocytes residing in large clusters found in the correlation network. Degree of synchronization for hepatocytes with their neighbors is frequently > = 3; **(D)** Average node degree distribution for large clusters. Nine out of Fourteen clusters show average degree > 2; **(E)** Clusters in correlation network mapped to their physical locations. Hepatocytes are represented as circles centered at their locations in the imaged field. Red stars mark the approximate locations of central veins (CV) in the imaged area. Hepatocytes belonging to a cluster have been plotted in the same color. Synchronized “islands” of hepatocytes cover only small regions of the imaged area.

Our data suggested an overall synchronization of intracellular Ca^2+^ dynamics across all 1300 hepatocytes that were segmented within the imaged field even though these hepatocytes were often separated by several cell layers. With a correlation-based network analysis, we sought to identify the typical spatial range within with Ca^2+^ responses of individual hepatocytes are synchronized under the experimental conditions. The correlation networks were constructed using pairwise Spearman rank correlation coefficients between Ca^2+^ traces. We used a minimum correlation coefficient cutoff (R_th_) of 0.75 and maximum inter-hepatocyte distance cutoff (d_th_) of 100 μm to assign network edges between two hepatocytes. The resulting network consisted of 669 hepatocytes with 565 edges between them. The node degree distribution for all hepatocytes in the network suggested that a large number of hepatocytes were synchronized with one or two other hepatocytes, for the chosen R_th_ and d_th_ values (Figure 2A). In order to identify the typical spatial extent of Ca^2+^ response synchronization among hepatocytes, we decomposed the network into isolated clusters. We found a set of 14 clusters containing more than 8 hepatocytes in each cluster (Figure 2B).

We focused our analysis on clusters that consisted of at least 8 hepatocytes (herein referred to as large clusters) for further analysis. For each large cluster, we estimated node degrees for individual hepatocytes as well as the average node degree for all hepatocytes within the cluster. Node degrees of individual hepatocytes residing in large clusters ranged between 1 and 7 (Figure 2C). Eighty seven out of the 190 hepatocytes residing in the large clusters had node degrees 3 or greater. Nine out of the 14 large clusters exhibited average node degrees > 2 (Figure 2D).

Mapping of the large clusters onto their physical locations (Figure 2E) suggested the existence of “islands” of synchronized Ca^2+^ response, which were generally situated close to the central veins. The typical spatial dimension of these synchronized clusters was less than the lobular dimensions (considered to be half the typical distance between approximate locations of two central veins). It must be noted that other smaller clusters, which are not shown in the figure, may be present in the intermittent space between the larger clusters.

**Figure 3:**
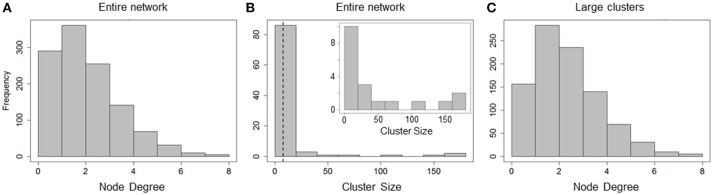
Cluster sizes and degree distribution for causal network. **(A)** Total node degree distribution of all hepatocytes in the network; **(B)** Cluster size distribution of all and large clusters (clusters with sizes > = 8, inset). Cluster sizes are much higher than those in correlation network analysis; **(C)** Total node degree distribution for all hepatocytes in large clusters. Hepatocytes are causally connected to up to 8 neighbors.

In summary, analysis of correlation networks constructed based on hepatocyte Ca^2+^ response showed that, despite an apparent global concurrence of Ca^2+^ peak intensities, synchronized hepatocytes formed localized clusters spanning small regions within liver lobules. Ca^2+^ responses of up to 7 hepatocytes were synchronized within these clusters. However, only a small fraction of hepatocytes was included in clusters with sizes 8 or greater (Figure 3C) suggesting that a correlation-based formulation of cell-cell interactions is insufficient to explain the observed lobular scale propagation of Ca^2+^ waves.

### Causal network analysis

We constructed causal networks between hepatocytes to identify whether adjacent neighbor-driven intercellular interactions can account for lobular scale Ca^2+^ wave propagation. We used a cell-oriented, transfer entropy (TE) based approach to identify causal connectivity between neighboring hepatocytes (see Methods). We considered molecular exchange between hepatocytes as the basis of causal influence between spatially co-localized hepatocytes and therefore allowed causal edges to exist only between closest neighbors. In our analysis, a unidirectional alignment of causal edges would suggest an organized, wave-like information flow along hepatocytes. We analyzed the resulting causal network for average node degree, total node degree, in and out node degrees, and direction of causal edges, to identify how many neighbors typically influence a given hepatocyte and the directional orientation of cell-cell interactions.

The causal network comprised of 1,162 hepatocytes with 1,491 edges between them. The number of hepatocytes included in the causal network far exceeded that in the correlation network (669 hepatocytes with 565 edges) suggesting that causality analysis describes intercellular interactions between neighboring hepatocytes better than a correlation-based analysis. We analyzed total node degree and cluster sizes for all nodes in the graph. The total node degree distribution of the all hepatocytes in the network peaked at a value of 2, pointing to causal connections between a given hepatocyte and multiple neighbors (Figure 3A). Upon decomposing the causal network into isolated clusters, we found 19 large clusters (cluster size ≥ 8). However, unlike the large clusters in the correlation network, large clusters in the causal network consisted of a higher number of hepatocytes (up to 160 hepatocytes, Figure 3B). The total node degree distribution for all hepatocytes residing in large clusters (sizes ≥ 8) exhibited similar causal connectivity characteristics between hepatocytes and their neighbors (Figure 3C).

**Figure 4:**
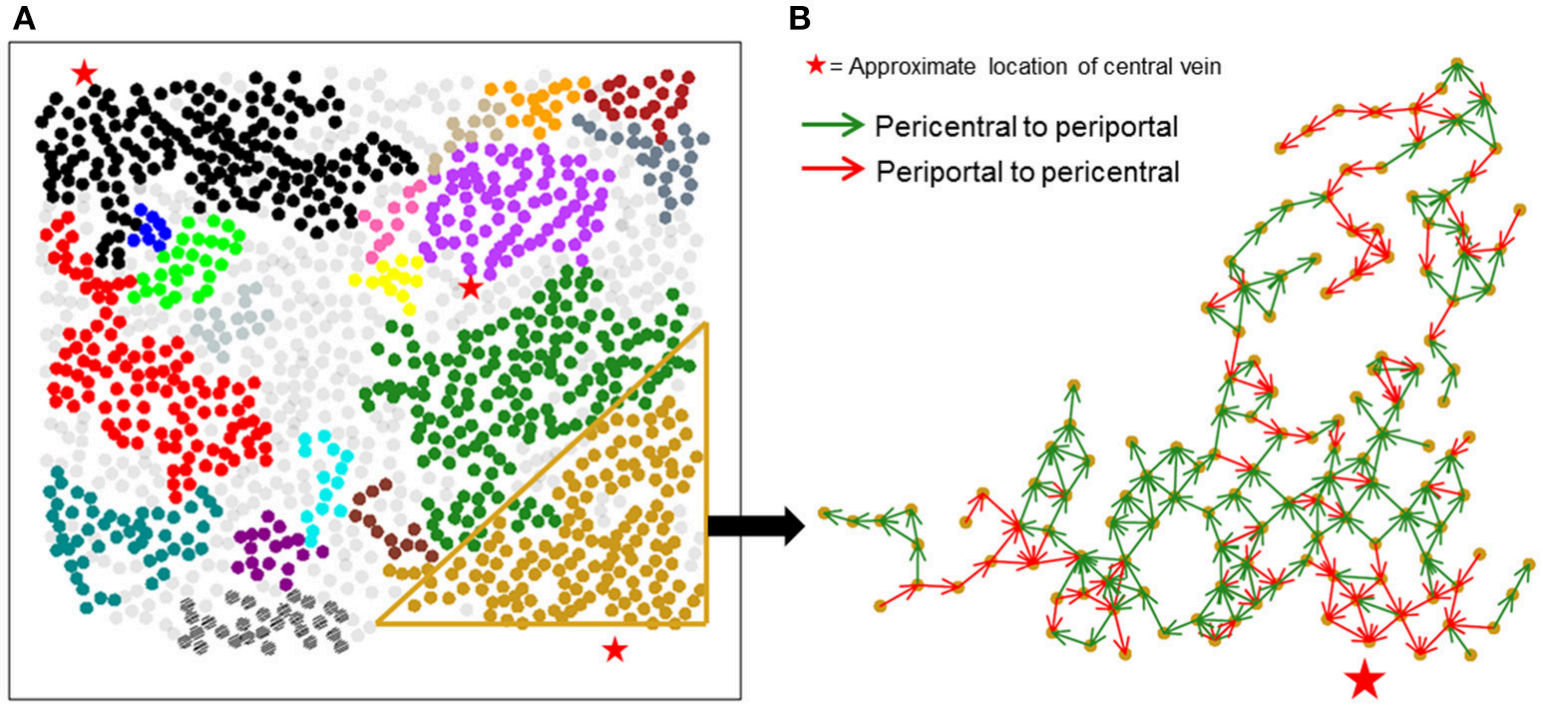
Spatial mapping of large causal networks. **(A)** Each cluster is represented by a different color. Large clusters (sizes > = 8) in causal network include 71.4% of the segmented hepatocytes (929 out of 1,300 hepatocytes); **(B)** causal influence edges in a representative cluster. Green arrows represent causal edges from the pericentral to the periportal region, whereas red arrows represent causal edges from the periportal region to the pericentral region. Causal influences identified between hepatocyte pairs are not aligned in a unidirectional fashion in the expected pericentral to periportal direction.

Figure 4A shows large isolated clusters in the causal network mapped to their physical locations within the imaged slice. The large clusters contain 929 of the 1,300 hepatocytes in the imaged area. In contrast to the correlation network, large clusters within the causal network span much larger areas of lobules compared to correlation network clusters. A visualization of the direction of causal influence between hepatocytes residing in a large cluster is shown in Figure 4B. Our analysis suggested that although hepatocytes were causally connected with a number of neighbors ranging from 1 to 8, the direction of causal influence was not consistently from the pericentral region to the periportal region.

### Computational model-based analysis of spatial Ca^2+^ wave propagation patterns

**Figure 5:**
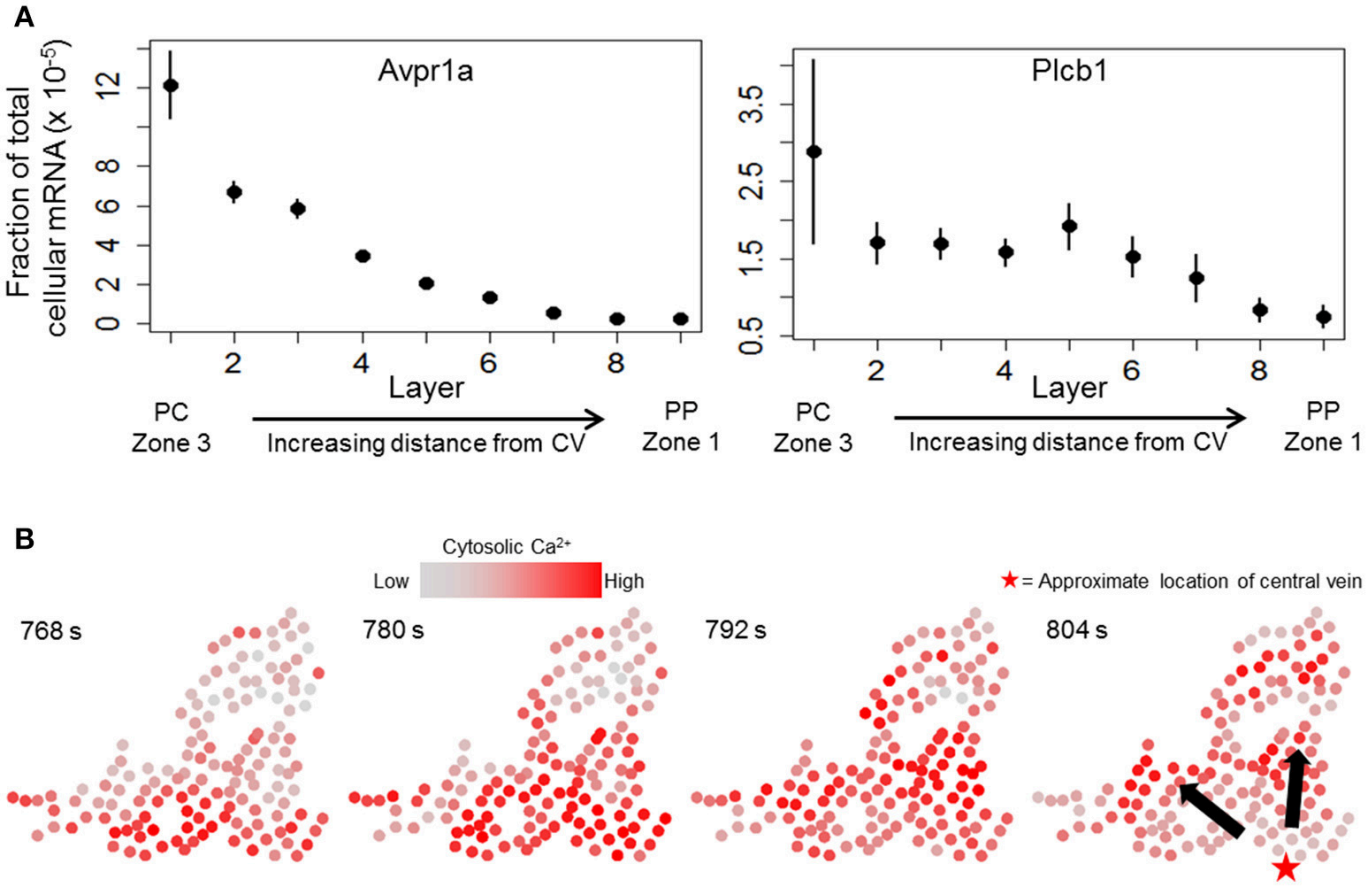
Ca^2+^ waves in experimental data. **(A)** zonation of Avpr1a (arginine—vasopressin receptor 1-a) and Plcb1 (PLC beta 1, linked to IP_3_ production rate) gene expression profiles across liver lobules (data from Halpern et al., 2017). Means and standard errors for gene expression values (fraction of total cellular mRNA) of each gene across the 9 layers show decreasing expression patterns from the pericentral region (PC) toward the periportal region (PP). Layer 1 lies closest to a central vein (acinar zone 3) whereas layer 9 is the farthest away (acinar zone 1); **(B)** Ca^2+^ wave propagation through a cluster identified using TE-based analysis. Ca^2+^ waves are initiated by hepatocytes lying close to the bottom of each panel and propagate upward. The red star in the right panel represents the approximate location of the central vein closest to the cluster. Ca^2+^ waves start from multiple hepatocytes residing near the bottom of each panel.

We next sought to determine whether a combination of the causal influence network and a dynamic model of hepatocyte Ca^2+^ response can yield propagating Ca^2+^ waves consistent with experimental observations. We started with a previously-published dynamic model of hepatocyte Ca^2+^ response (Verma et al., 2016) and extended the model to incorporate cell-cell connectivity suggested by the causal influence network (see Methods). In addition, we modified the model parameters to incorporate zonation patterns of signaling components based on results from recently published single cell RNA-seq study (Halpern et al., 2017). We mined the transcriptomic data set (Table S3 from Halpern et al., 2017) to identify zonation of mRNA expression of Ca^2+^ signaling relevant genes. Specifically, we considered the zonation patterns of arginine-vasopressin receptor 1a (Avpr1a) and Phospholipase C β-1 (Plcb1). Zonation profiles for Avpr1a and Plcb1 in the data from (Halpern et al., 2017) are shown in Figure 5A. Avpr1a expression levels and Plcb1 expression levels correspond to vasopressin receptor recycling rate (model parameter k_r_), and IP_3_ synthesis rate (model parameter k_IP3_), respectively, in the present dynamic model. For the subsequent analysis using integrated causal network and dynamic modeling, we considered a large cluster of hepatocytes identified using the causal network analysis. Experimentally determined Ca^2+^ patterns in this cluster of hepatocytes are shown in Figure 5B. Notable features of Ca^2+^ wave propagation through the cluster were: (1) Ca^2+^ waves propagated through the cluster from the pericentral region toward the periportal region consistent with the prior expectation, and (2) Ca^2+^ waves started from multiple hepatocytes located closer to the approximate location of the central vein residing closest to the cluster. We evaluated the dynamic model of this large cluster to identify the spatial patterns of intracellular signaling components as well as gap junction connectivity patterns that are consistent with experimentally observed Ca^2+^ wave propagation. In the dynamic model, Ca^2+^ response coupling is caused by gap junction mediated IP_3_ exchange, a phenomenon that has been reported previously (Tordjmann et al., 1997; Eugenín et al., 1998).

We simulated the dynamic model to identify the effect of gap junctions on coupling of Ca^2+^ dynamics across hepatocytes. Simulations were performed using the spatial locations of hepatocytes for the cluster shown in Figure 5B. The connectivity structure of the causal influence network from the TE-based analysis was utilized as the adjacency matrix for cell-cell IP_3_ exchange. We considered two modes of gap junction conductivity (model parameter G_ij_): (1) no hepatocyte exchanges IP_3_ with its neighbors, and (2) each hepatocyte exchanges IP_3_ with all its neighbors. Gap junction conductivity parameter between any pair of hepatocytes, modeled as a mass transfer term assuming fast IP_3_ diffusion kinetics, was set to either 0 (no IP_3_ exchange) or 0.9.

**Figure 6:**
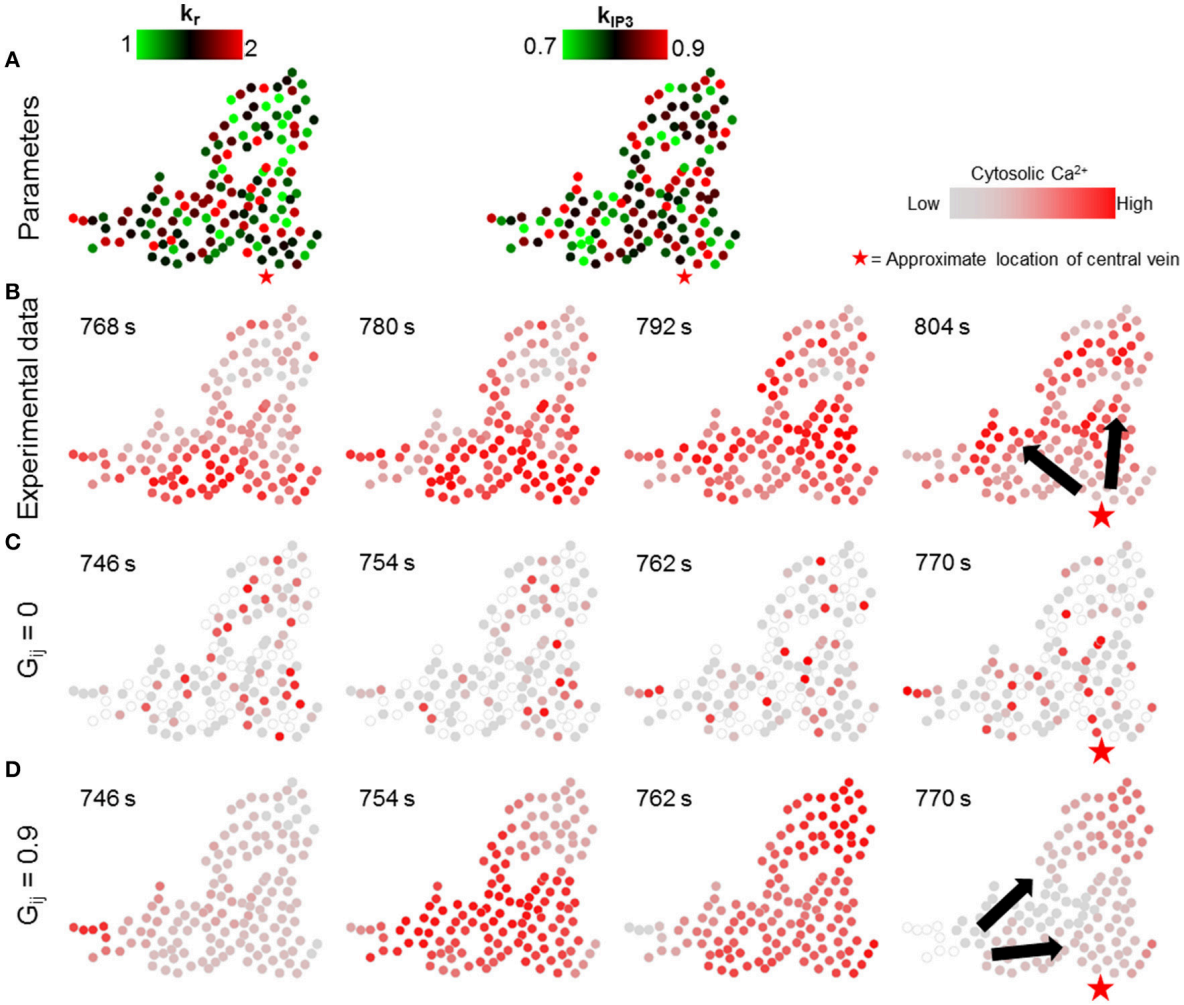
Effect of randomness of intracellular Ca^2+^ signaling parameters. Intracellular parameter values were randomly chosen from uniform distributions for each hepatocyte in the simulation (k_r_ ϵ [1, 2], k_IP3_ ϵ [0.7, 0.9]). **(A)** k_r_ and k_IP3_ values used for each hepatocyte; **(B)** Ca^2+^ waves in experimental data; **(C)** no gap junction mediated IP_3_ exchange; and **(D)** gap junction mediated IP_3_ exchange with all neighbors. With gap junctions switched off, there are no Ca^2+^ waves through the cluster **(C)**. With gap junctions switched on **(D)**, Ca^2+^ waves propagate, however, the direction of propagation is not consistent with that observed experimentally. The red stars show the approximate location of the closest central vein. Note that the time points shown in each case were selected to best depict Ca^2+^ dynamics and spatial propagation. The times shown in **(B)** are with reference to the experiment start time. Times shown in **(C,D)** were measured from the time when stimulus was introduced in the simulations (*T* = 200 s).

In the first set of simulations, the individual hepatocyte-specific values of signaling parameters k_r_ and k_IP3_ were sampled from uniform distributions over nominal parameter ranges listed in Verma et al. (2016) (k_r_ ϵ [1, 2] s^−1^, k_IP3_ ϵ [0.7, 0.9] × 10^4^ μMs^−1^, Figure 6A). The corresponding simulation results demonstrate that lobular scale Ca^2+^ waves did not occur when the gap junction-mediated IP_3_ exchange was turned off (Figure 6C). By contrast, Ca^2+^ waves propagated through the cluster, when each of the hepatocytes exchanged IP_3_ with all their neighbors (Figure 6D). However, the direction of wave propagation was not necessarily consistent with the experimental observations (Figure 6B).

**Figure 7:**
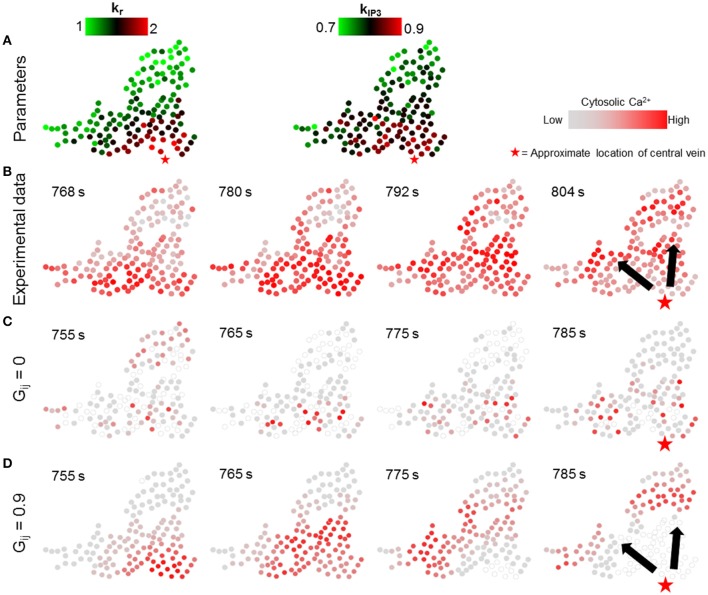
Effect of lobular gradients of intracellular Ca^2+^ signaling parameters. **(A)** k_r_ and k_IP3_ values used for each hepatocyte. k_r_ values decay exponentially with increase in the distance of a hepatocyte from the central vein whereas k_IP3_ values decrease linearly with increasing distance from the central vein; **(B)** Ca^2+^ waves in experimental data; **(C)** no gap junction mediated IP_3_ exchange; and **(D)** gap junction mediated IP_3_ exchange with all neighbors. No Ca^2+^ waves appear when gap junctions are switched off **(C)**. With gap junctions switched on **(D)** Ca^2+^ waves propagate through the cluster. However, the Ca^2+^ waves start from only a few hepatocytes. A Ca^2+^ wave propagates through the cluster in ~ 30 s, as compared to ~ 50 s in the experiment. The red stars show the approximate location of the closest central vein. Note that the time points shown in each case were selected to best depict Ca^2+^ dynamics and spatial propagation. The times shown in **(B)** are with reference to the experiment start time. Times shown in **(C,D)** were measured from the time when stimulus was introduced in the simulations (*T* = 200 s).

We next simulated the dynamic model with the values of parameters k_r_ and k_IP3_ drawn from spatial profiles that mimicked the zonated gene expression levels observed experimentally in Halpern et al. (2017). We approximated spatial profiles for mean k_r_ and k_IP3_ values as exponentially and linearly decreasing functions with increasing distance from central vein, respectively, confined within the nominal parameter ranges (Figure 7A; Table 1; Verma et al., 2016). Parameter values for all hepatocytes in the model were initialized based on their distance from the central vein with additive noise (see Figure S6 for a description of model parametrization). We evaluated the effect of changing gap junction conductivity, according to the two modes considered in simulations shown in Figure 6.

The simulation results suggested that even with gradients in parameters governing receptor-mediated signaling and IP_3_ synthesis, Ca^2+^ waves did not arise in the absence of molecular exchange (Figure 7C). In the simulations, propagating Ca^2+^ wave patterns consistent with experiments were observed when hepatocytes exchanged IP_3_ with their neighbors (Figure 7B, D). Our simulation results differed from experiments with regards to the region where Ca^2+^ waves are initiated. In the experimental observations, Ca^2+^ waves started from hepatocytes spread out in a relatively wider area close to the central vein. This difference could be due to the fact that in our dynamic model, hepatocytes were parametrized based on their distance from a central vein approximated as a point, when in reality the pericentral hepatocyte phenotype might result from microenvironmental signals in a more diffused region surrounding the central veins, whose diameters could span a few cell layers.

**Figure 8:**
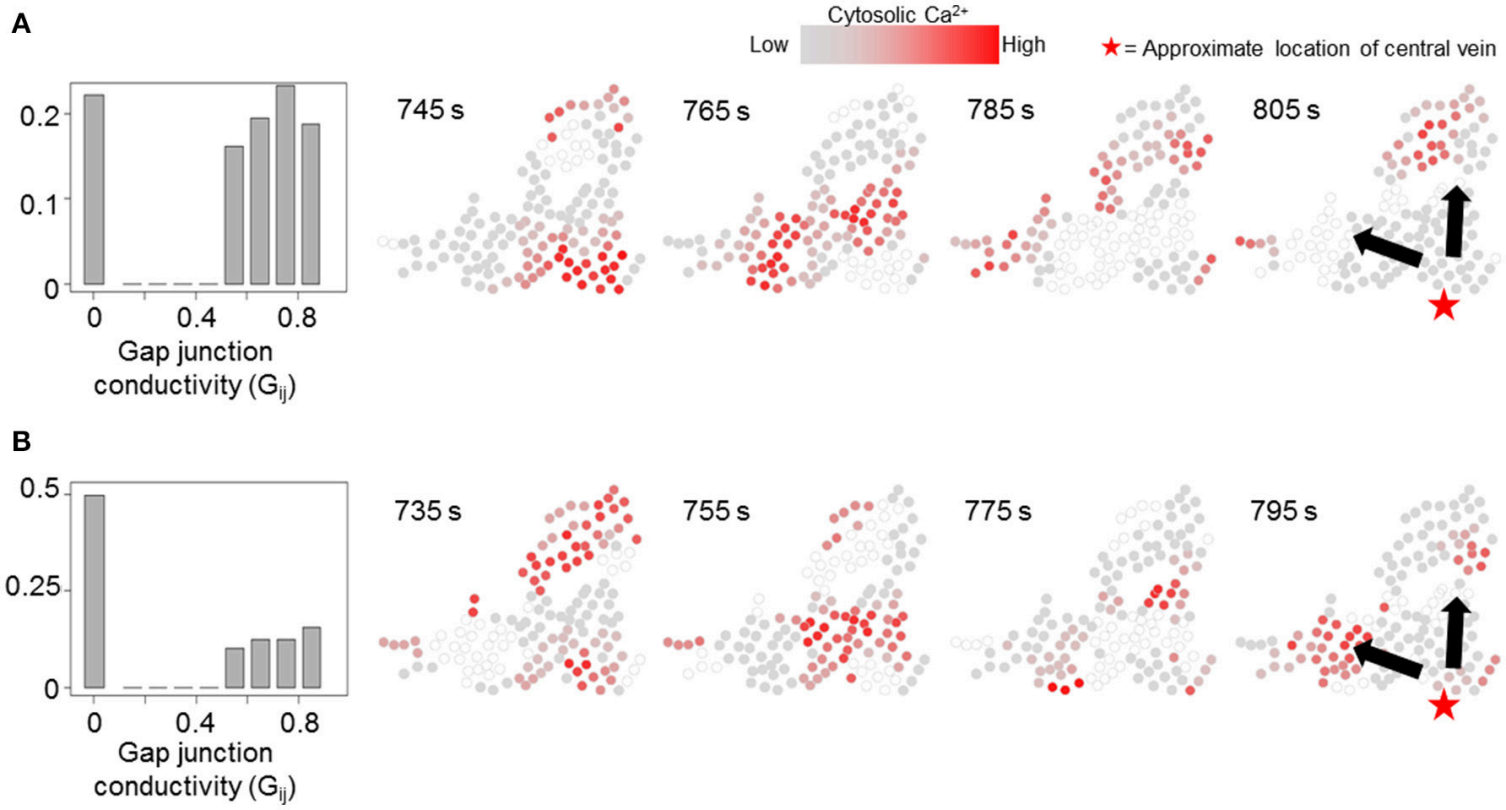
Effect of non-uniform gap junction conductivity on Ca^2^+ signal propagation. Normalized G_ij_ value histograms and Ca^2+^ wave propagation with 20% (p_th_ = 0.2, **A**) and 50% (p_th_ = 0.5, **B**) likelihood of a given IP_3_ mass transfer term (G_ij_) being 0. 22.1% G_ij_ values in **(A)** and 49.6% G_ij_ values in **(B)** are 0. In either case, Ca^2+^ waves propagate through the cluster. Note that the time points shown in each case were selected to best depict Ca^2+^ dynamics and spatial propagation. Times shown in **(A,B)** were measured from the time when stimulus was introduced in the simulations (*T* = 200 s).

We simulated our model to identify the effects of non-homogeneous gap junction conductivity by varying parameter G_ij_. Heterogeneity in gap junction conductivity could account for variability in cell-cell contact areas and gap junctions themselves. We considered two modes of gap-junction non-uniformity where either 20 or 50% G_ij_ values were likely to be 0 to account for a fraction of hepatocyte pairs not interacting with each other. Additionally, the non-zero G_ij_ values in either case were randomly drawn from a uniform distribution [ϵ [0.5, 0.9]] to account for variability in gap junction conductivity and number between a pair of adjacent hepatocytes. Other cell-intrinsic parameter values were identical to those used in the simulations corresponding to Figure 7. Effect of gap junction heterogeneity on spatial patterns of Ca^2+^ signal propagation through a cluster of hepatocytes identified using the TE-based analysis are shown in Figure 8 (see Figure S7 for cell-cell connections). We observed that in our simulations Ca^2+^ waves propagated through the cluster despite 22.1% (Figure 8A) and 50% (Figure 8B) G_ij_ values set to 0. Consistent with expectation, Ca^2+^ waves propagated in the direction of intracellular parameter gradients in both cases. Our simulations suggested that multiplicity of hepatocyte interactions makes Ca^2+^ wave propagation robust to non-interacting hepatocyte pairs.

Since the store Ca^2+^ concentration is nearly 1000 times higher than cytosolic Ca^2+^ concentration we considered an alternative model in which the store Ca^2+^ was considered to be a constant (see section Methods). In this model formulation, store Ca^2+^ could be interpreted as a driving force for influx of Ca^2+^ within the cytosol instead of being trafficked between the cytosol and the Ca^2+^ store. Ca^2+^ wave propagation characteristics in this case were similar to the case in which store Ca^2+^ was dynamic (see Figure S4).

### Low vs. high stimulus

We used a step-wise increasing vasopressin stimulus over the course of the live tissue imaging duration (Figure 1). To identify the effects of change in stimulus, we compared the induced correlation and causal networks present at different stimulus levels in our data. We divided the time course into low and high stimulus regimes based on overall increase in rates of cytosolic Ca^2+^ spikes and compared the cluster sizes and localization for correlation and causal networks (Figures S8–S10). Our analysis revealed that although there was an increase in the number of large clusters in the high stimulus regime, the size of the clusters decreased (Figure S8). In contrast, we found similar large clusters in the high stimulus regime with a moderate increase in cluster sizes present in the causal network. In either case, regions of the image spanned by the large clusters were dependent on the stimulus level (Figure S9).

## Discussion

In this work, we analyzed Ca^2+^ signal propagation in a two-dimensional optical slice of a perfused and intact mouse liver at the lobular scale. We generated a large-scale data set on cytosolic Ca^2+^ responses of 1300 hepatocytes to hormonal stimuli over a period of 1600 s. We analyzed the synchronization of Ca^2+^ response of a large population of hepatocytes in intact liver using correlation analysis and TE-based causal network analysis to identify directional flow of causal influence across hepatocytes in a lobule. We employed a computational model-based analysis to identify spatial patterns of intracellular Ca^2+^ signaling components and gap junction conductivity that can yield lobular scale Ca^2+^ waves consistent with experimental observations.

Identification of functional networks within a population of colocalized cells has gained prominence in recent decades (Bullmore and Sporns, 2009; Ahrens et al., 2013; Tian et al., 2018). Correlation (Fox et al., 2005; StoŽer et al., 2013; Markovič et al., 2015) as well as causal (Lungarella and Sporns, 2006; Wollstadt et al., 2014; Seth et al., 2015) network analysis are viable strategies to identify functional connectivity in cell populations. Correlation networks are commonly used to analyze Ca^2+^ responses in a population of cells under a global stimulus. For instance, correlation networks constructed using Ca^2+^ dynamics in Islets of Langerhans exhibit stimulus-dependent synchronization characteristics when stimulated by glucose (StoŽer et al., 2013; Markovič et al., 2015). However, correlation network analysis was insufficient to explain lobular scale propagation of Ca^2+^ waves observed in our experiment. In contrast, causal network analysis of the experimental data elucidated prominent features of lobular scale Ca^2+^ wave propagation such as existence of “islands” of causally connected hepatocytes within liver lobules and lack of directional alignment of causal edges between hepatocytes from the pericentral region to the periportal region. Although causal network analysis yielded misaligned causal connections between hepatocytes residing in a cluster, it pointed toward zonation and intercellular communication as cell-level dynamics that yield lobular scale organization of Ca^2+^ response. Spatially organized heterogeneity leads to location-based differences in Ca^2+^ signaling capacity of hepatocytes. Intercellular communication results in entrainment of Ca^2+^ responses of adjacent hepatocytes which extends throughout liver lobules via local interactions to yield Ca^2+^ waves.

The computational model of Ca^2+^ dynamics used in our study is limited in its scope. Our dynamic model-based analysis, parametrized using lobular scale spatial patterns of liver gene expression, represents a specific case of a more generalized concept of functional gradients that control Ca^2+^ wave propagation in liver lobules. Functional zonation results from zonal differences in micro-RNA expression (Sekine et al., 2009; Chen and Verfaillie, 2014) as well as protein activity (Gebhardt and Mecke, 1983; Jungermann, 1987). Cytosolic Ca^2+^ spiking dynamics have previously been observed in rat hepatocytes due to activation of adrenergic (Rooney et al., 1990) and purinergic (Dixon et al., 1990) receptors. Of the wide array of cell surface receptors and extracellular stimuli that could be spatially organized in liver lobules, our model-based analysis considered zonation of Avpr1a and Plcβ1 only, and response to hormone-induced GPCR signaling cascade. Additionally, our deterministic model ignores stochasticity in cellular level phenomena. For example, we modeled gap junction mediated molecular exchange as a flux term wherein channel conductivity between a pair of hepatocytes remained constant over time. However, a probabilistic treatment of open and closed channels, possibly linked to intracellular Ca^2+^ signaling events (Török et al., 1997; Peracchia, 2004; De Vuyst et al., 2006), may capture cell-neighbor molecular interactions more accurately. Explicit consideration of a comprehensive intracellular Ca^2+^ signaling cascade with zonal information, a stochastic modeling framework, and integration of experimental data can potentially capture the complexity observed in lobular scale Ca^2+^ dynamics in the liver, such as lack of directionality of causal linkages between hepatocytes.

Although Ca^2+^ as well as IP_3_ could be exchanged between neighboring hepatocytes through gap junctions and lead to Ca^2+^ efflux from intracellular stores, the effective diffusivity of IP_3_ is higher than Ca^2+^ because Ca^2+^ is heavily buffered within hepatocytes (Allbritton et al., 1992). These observations suggest that IP_3_ is strongly involved in coordinating Ca^2+^ responses at the lobular scale. In addition, a loss of wave-like propagation of Ca^2+^ signals has been shown upon disruption of cell-cell contacts using Ca^2+^ free buffer (Gaspers and Thomas, 2005). Intracellular Ca^2+^ mobilization could arise from paracrine ATP release and subsequent purinergic receptor activation. The relative contribution of Ca^2+^ response synchronization via gap junctions or paracrine ATP would depend on the tissue and zone-specific expression of Connexin subtypes and purinergic receptors. Disrupting cell-cell contacts between hepatocytes in perfused livers results in asynchronous Ca^2+^ spikes in hepatocytes under a vasopressin stimulus and the Ca^2+^ signals do not spread to neighbors (Gaspers and Thomas, 2005). These results suggest that the paracrine ATP release is not sufficient to drive a lobular scale Ca^2+^ signal propagation observed experimentally. That said, explicit consideration of other potential paracrine factors such as ATP will expand the scope and applicability to time scales of cell-cell interaction beyond the relatively fast timescale considered in this study.

We note that a variety of fluorophores are available for reporting intracellular calcium levels, including genetically encoded calcium reporters. For example, Fluo-8 AM and Rhod4 have been utilized for cytosolic calcium reporting in hepatocytes with small differences in Kd values and photostability (Lock et al., 2015). We have been using Fluo-8 AM with good success in previous studies (Bartlett et al., 2017) and therefore utilized this reporter for obtaining the dynamic data analyzed in the present study.

An important consideration in analyzing and modeling Ca^2+^ signal propagation within liver lobules is the three-dimensional arrangement of hepatocytes. Although the proximity of hepatocytes to either a portal triad or a central vein within a two-dimensional slice can be ascertained, information regarding the cellular adjacency and spatial localization along a third dimension is lacking in our experimental data. The lack of directional alignment of causal edges along a pericentral to periportal orientation could arise due to the presence of micro-environmental cues from other pericentral or periportal regions above or below the optical slice corresponding to the imaged area. Alternatively, multidirectional alignment of causal edges may be due to cell-autonomous Ca^2+^ responses of hepatocytes within a small region which appear independently of the global stimulus and do not propagate beyond a few cells. High spatial and temporal resolution imaging of three-dimensional tissue structure sufficient to study spatial organization of Ca^2+^ signaling in liver lobules remains a challenging problem. However, imaging techniques are constantly evolving to produce accurate three-dimensional reconstructions of tissues with high spatial resolution (Arganda-Carreras et al., 2010; Hoehme et al., 2010). Intra-vital imaging techniques for visualizing molecular dynamics in live animals (Benechet et al., 2017; Dunn and Ryan, 2017) could further augment our modeling efforts at small spatial scales. However, these methods would introduce new challenges such as lack of control over distribution of stimulus in the immediate microenvironment of hepatocytes. Application of a combination of three-dimensional reconstruction and intra-vital imaging may provide data with the high spatial and temporal resolution required for a detailed dynamic model-based accounting of Ca^2+^ signal propagation in liver lobules.

## Author contributions

AV, JH, and RV designed the study. AA conducted the experiments. AA, AN, JH, and RV analyzed experimental data and interpreted the results. AV performed causal network analysis. AV developed computational model and performed simulations. AV, BO, and RV analyzed and interpreted the network analysis and simulation results. AV and RV drafted the manuscript. AA, BO, JH, and RV edited the manuscript. JH supervised the experimental aspects. RV supervised the computational aspects.

### Conflict of interest statement

The authors declare that the research was conducted in the absence of any commercial or financial relationships that could be construed as a potential conflict of interest.
