# Supplementary figures and images for: Causality Analysis and Cell Network Modeling of Spatial Calcium Signaling Patterns in Liver Lobules
Source: Front Physiol. 2018 Oct 4;9:1377. doi: 10.3389/fphys.2018.01377 (PMC6180170; doi:10.3389/fphys.2018.01377)

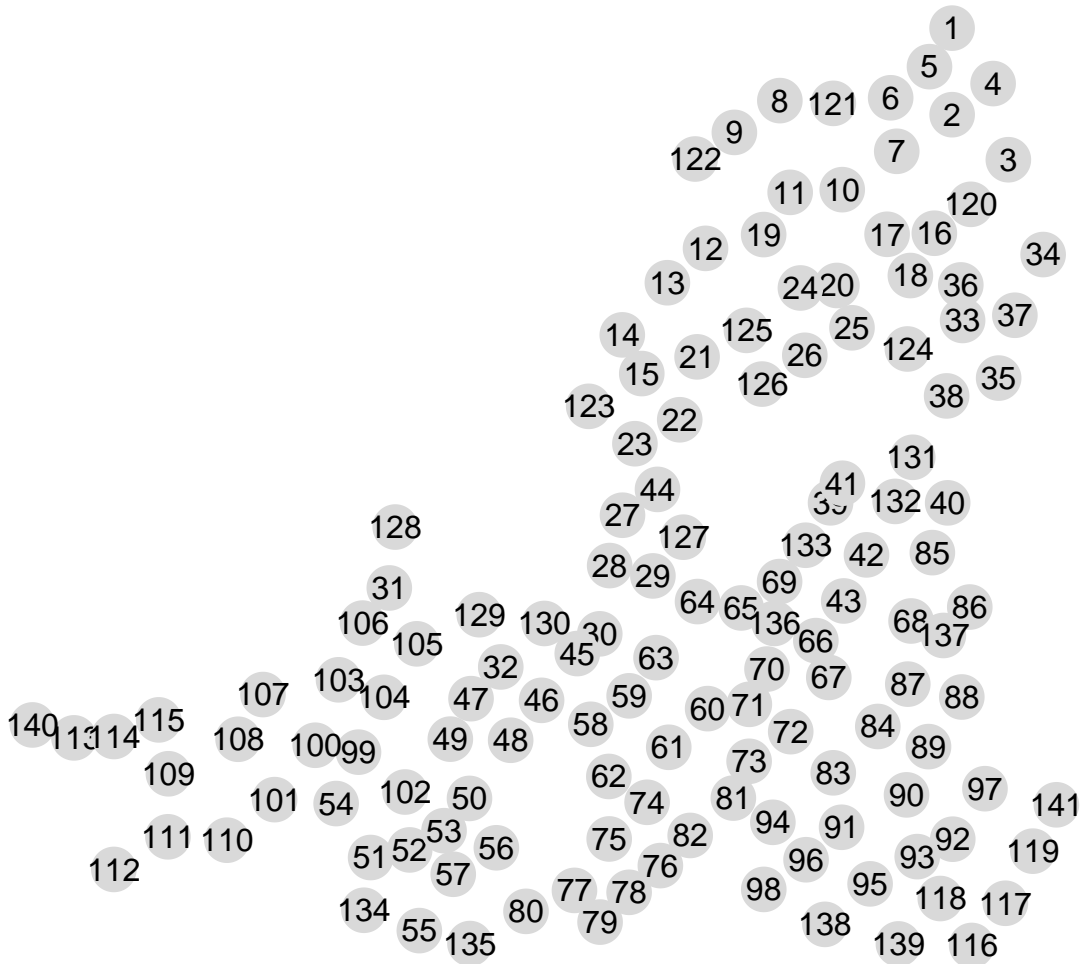

Supplement: Supplementary file 1 [file Data_Sheet_1.ZIP › Vadigepalli-Supplementary-Data-Code/Cell_aliases.pdf]
