# Supplementary material for: Causality Analysis and Cell Network Modeling of Spatial Calcium Signaling Patterns in Liver Lobules
Source: Front Physiol. 2018 Oct 4;9:1377. doi: 10.3389/fphys.2018.01377 (PMC6180170; doi:10.3389/fphys.2018.01377)
Supplement: Supplementary file 2 [file Image_1.PDF]

1

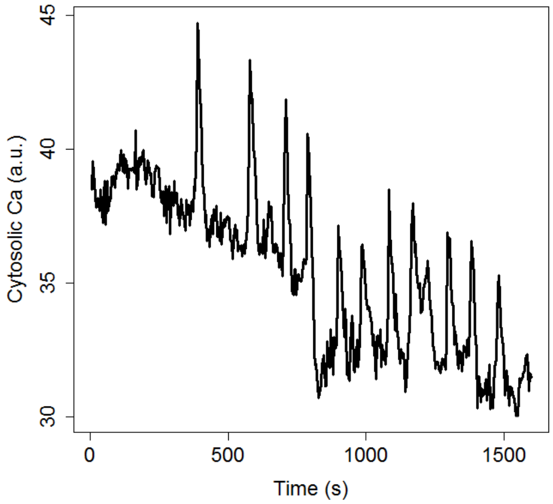

4-A

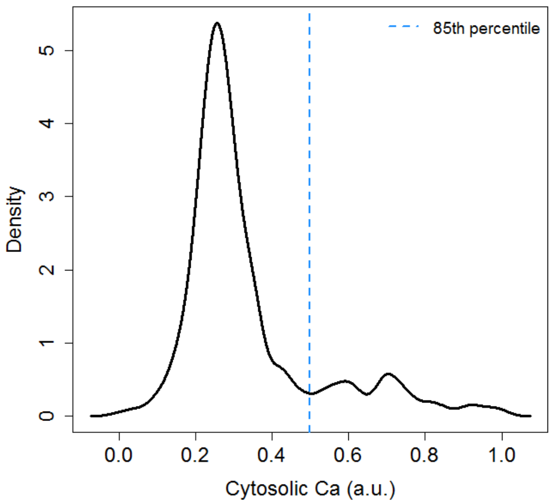

2

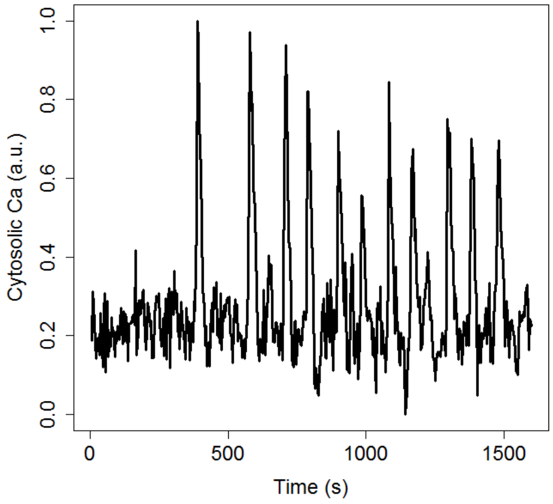

4-B

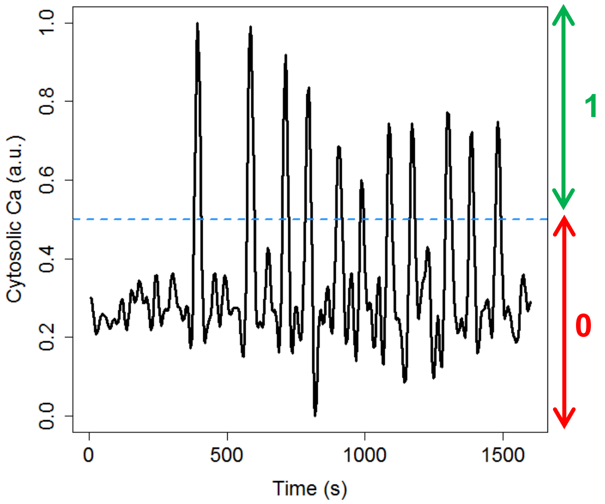

3

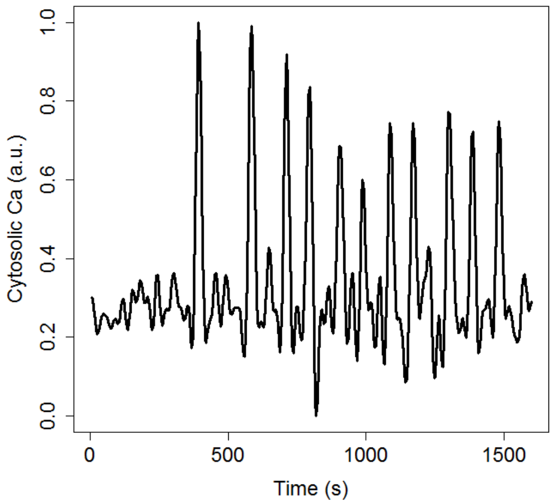

Figure S1: data preprocessing. 1 – Raw time cytosolic Ca^{2+} time trace for a hepatocyte; 2 – Baseline corrected and rescaled time trace. Baseline correction was performed using the baseline package (version 1.2) in R (version 3.2.3); 3 – Low pass filtered time trace. Baseline corrected and rescaled time traces were subjected to low pass filtering using the smooth.fft function from the itsmr package (version 1.5) in R (version 3.2.3). Only the lowest 27.5 percentile of the power spectrum was accepted in the filtered data; 4 – A: Quantization threshold for low pass filtered time trace. The 85th percentile intensity value over the entire time trace was used as a threshold to quantize the Ca^{2+} values at each time point as low (0) or high (1), as shown in 4-B.
