# Supplementary material for: Causality Analysis and Cell Network Modeling of Spatial Calcium Signaling Patterns in Liver Lobules
Source: Front Physiol. 2018 Oct 4;9:1377. doi: 10.3389/fphys.2018.01377 (PMC6180170; doi:10.3389/fphys.2018.01377)
Supplement: Supplementary file 3 [file Image_2.PDF]

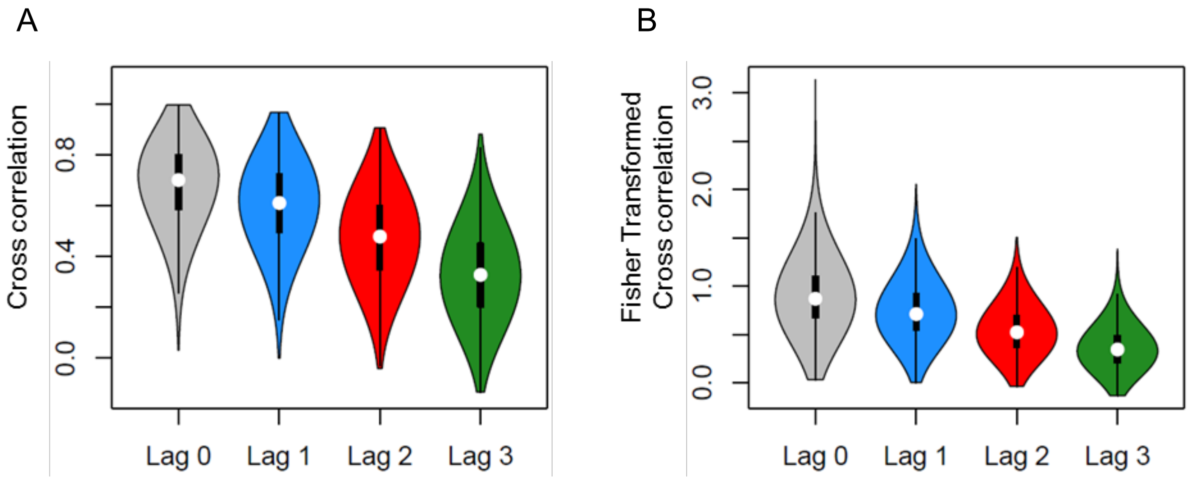


Figure S2: Raw (A) and Fisher transformed (B) cross correlation values between all hepatocyte and their neighbors. The neighbors for every hepatocyte were the same as those used in the TE-based causal analysis. Lags 0, 1, 2, and 3 correspond to time shifts of 0, 4, 8 and 12 seconds in during the experiment respectively. Although lag 0 correlation show the highest magnitudes, correlation based analysis does not capture lobular scale $Ca^{2+}$ waves. We selected a lag value of 1 for our TE-based analysis because of higher cross correlations between $Ca^{2+}$ responses of hepatocytes and their neighbors corresponding to a 4 second time delay.
