# Supplementary material for: Causality Analysis and Cell Network Modeling of Spatial Calcium Signaling Patterns in Liver Lobules
Source: Front Physiol. 2018 Oct 4;9:1377. doi: 10.3389/fphys.2018.01377 (PMC6180170; doi:10.3389/fphys.2018.01377)
Supplement: Supplementary file 4 [file Image_3.PDF]

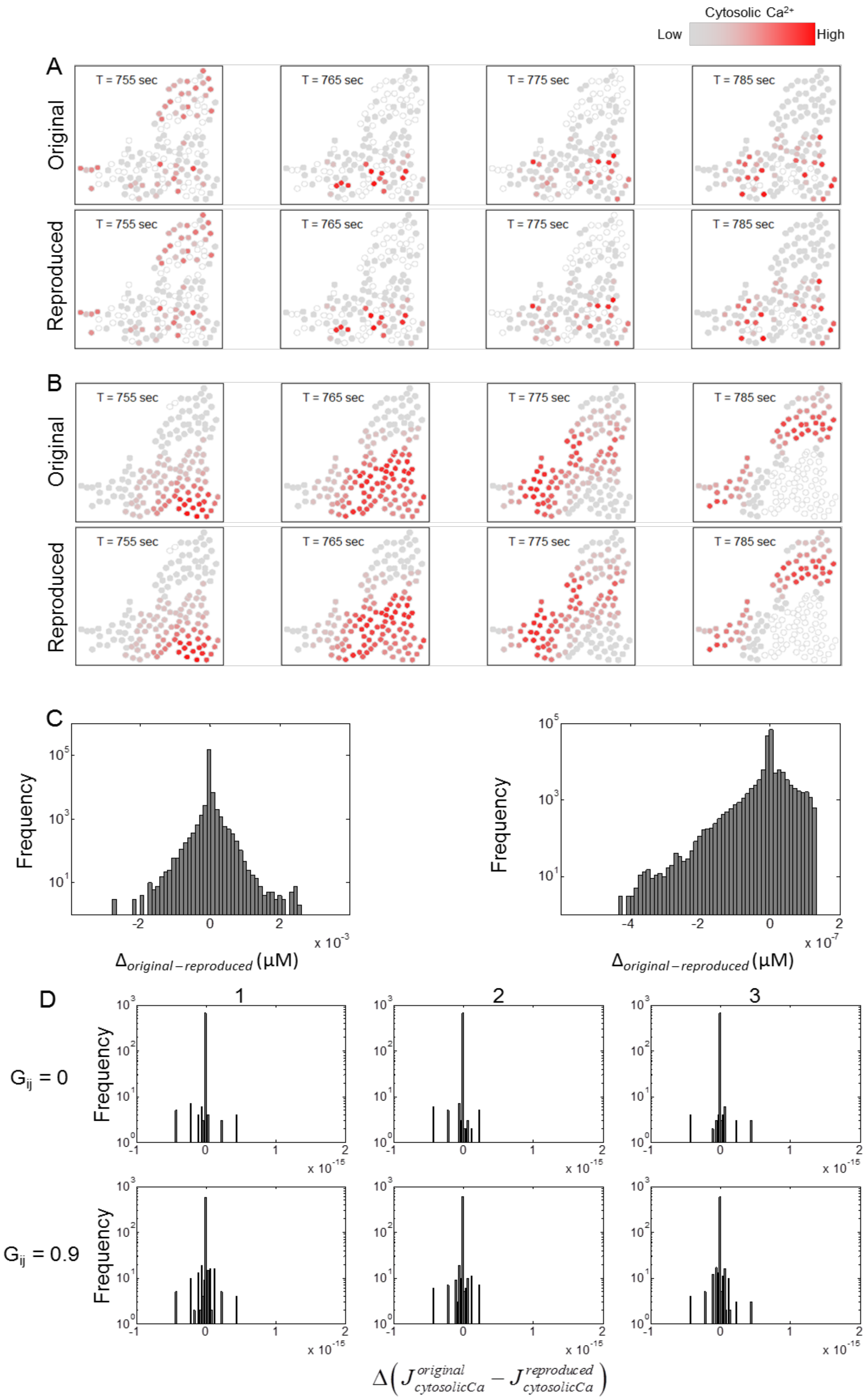

Cytosolic Ca2+
Low
High
A
Original
Reproduced
T = 755 sec
T = 765 sec
T = 775 sec
T = 785 sec
B
C
Frequency
Δoriginal−reproduced (μM)
x 10-3
x 10-7
D
1
2
3
Gij = 0
Gij = 0.9
x 10-15
Δ(J original cytosolicCa − J reproduced cytosolicCa)

Figure S3: Model reproducibility. Simulation results presented in this work were reproduced independently using the model equations in the main text, and the parameter values and adjacency matrix provided as supplementary material. In either case, transient cytosolic Ca^{2+} values were stored for a period of 1200 seconds at 1 second intervals. Stimulus was introduced (H = 1.8e-10 M) after 200 seconds. Values plotted are shown at time after introduction of stimulus. **A**: Original and reproduced cytosolic Ca^{2+} levels shown in Figure 7C. In this case intracellular parameters k_r and k_{IP3} were initialized following pericentral to periportal gradients but gap junctions were switched off. Values for the two parameters can be found in the file Fig6_params.txt. Simulation data presented in Figure 7C can be found in file Fig7C_data.txt; **B**: Original and reproduced cytosolic Ca^{2+} levels shown in Figure 7D. Parameter values used were same as those in Figure 7C. Simulation data presented in Figure 7D can be found in file Fig7D_data.txt; **C**: Histogram of the differences between original simulations and reproduced data corresponding to Figures 7C and 7D for 141 cells over a 1200 second simulation. **D**: Differences in rates of change of cytosolic Ca^{2+} between the original and reproduced models. The differences are shown for 3 randomly generated input vectors within the typical concentration ranges for the state variables. The rates estimated are in close agreement (differences on the order of 10^{-16} μM)
