# Supplementary material for: Causality Analysis and Cell Network Modeling of Spatial Calcium Signaling Patterns in Liver Lobules
Source: Front Physiol. 2018 Oct 4;9:1377. doi: 10.3389/fphys.2018.01377 (PMC6180170; doi:10.3389/fphys.2018.01377)
Supplement: Supplementary file 5 [file Image_4.PDF]

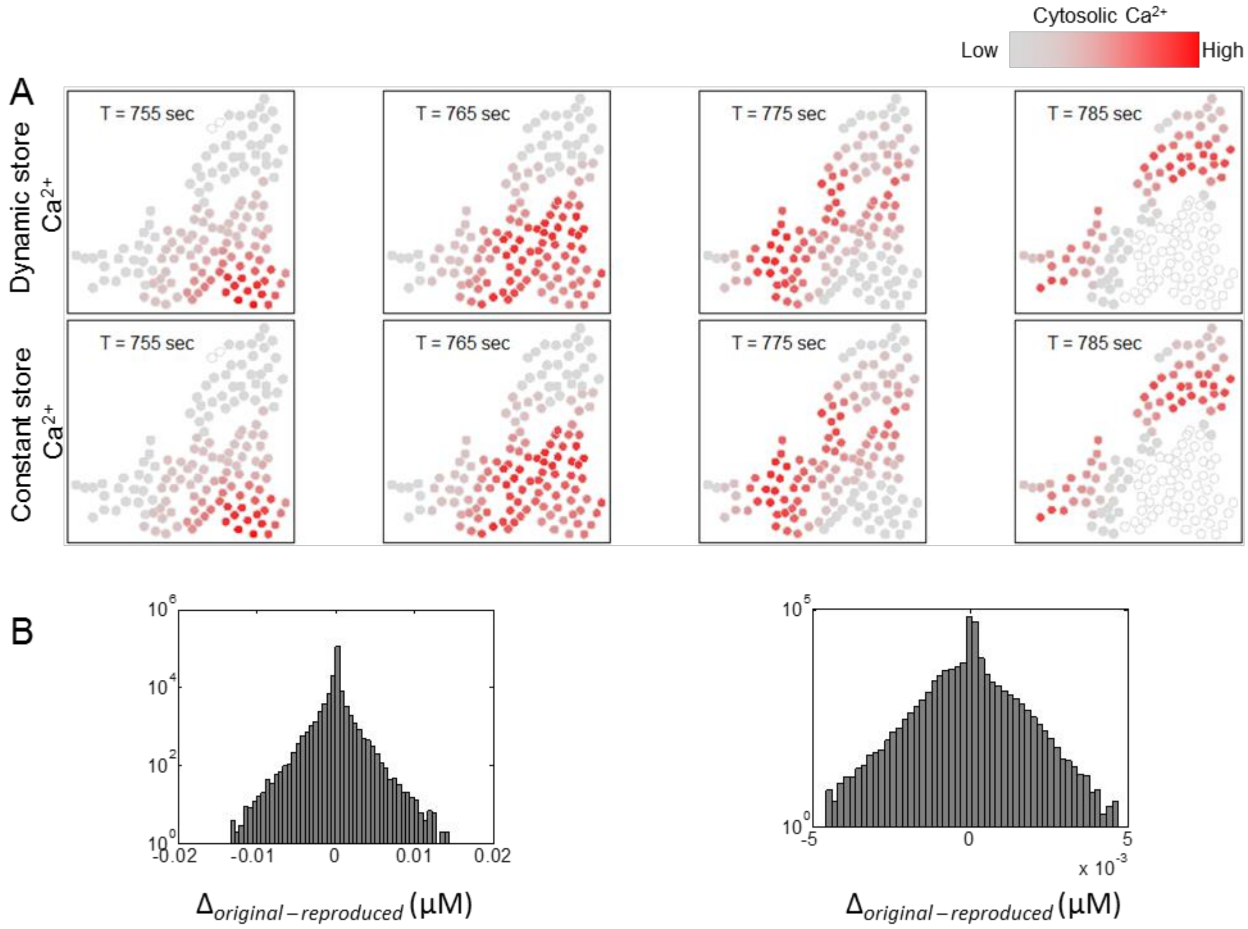

Figure S4: **A**: Alternative modeling scheme, where total Ca^{2+} in the hepatocyte is considered to be fixed at a constant value (= 500 μM). Since the total Ca^{2+} is nearly 1000 times higher than cytosolic Ca^{2+} it can be assumed to be a constant. Ca^{2+} wave propagation characteristics in this case are very similar to the original simulations. Although the wave propagation characteristics remain the same, the differences between cytosolic Ca^{2+} levels for 141 cells over a 1200 second simulation period range up to 2% of the typical Ca^{2+} amplitude (1 μM) , as shown in **B**.
