# Supplementary material for: Causality Analysis and Cell Network Modeling of Spatial Calcium Signaling Patterns in Liver Lobules
Source: Front Physiol. 2018 Oct 4;9:1377. doi: 10.3389/fphys.2018.01377 (PMC6180170; doi:10.3389/fphys.2018.01377)
Supplement: Supplementary file 6 [file Image_5.PDF]

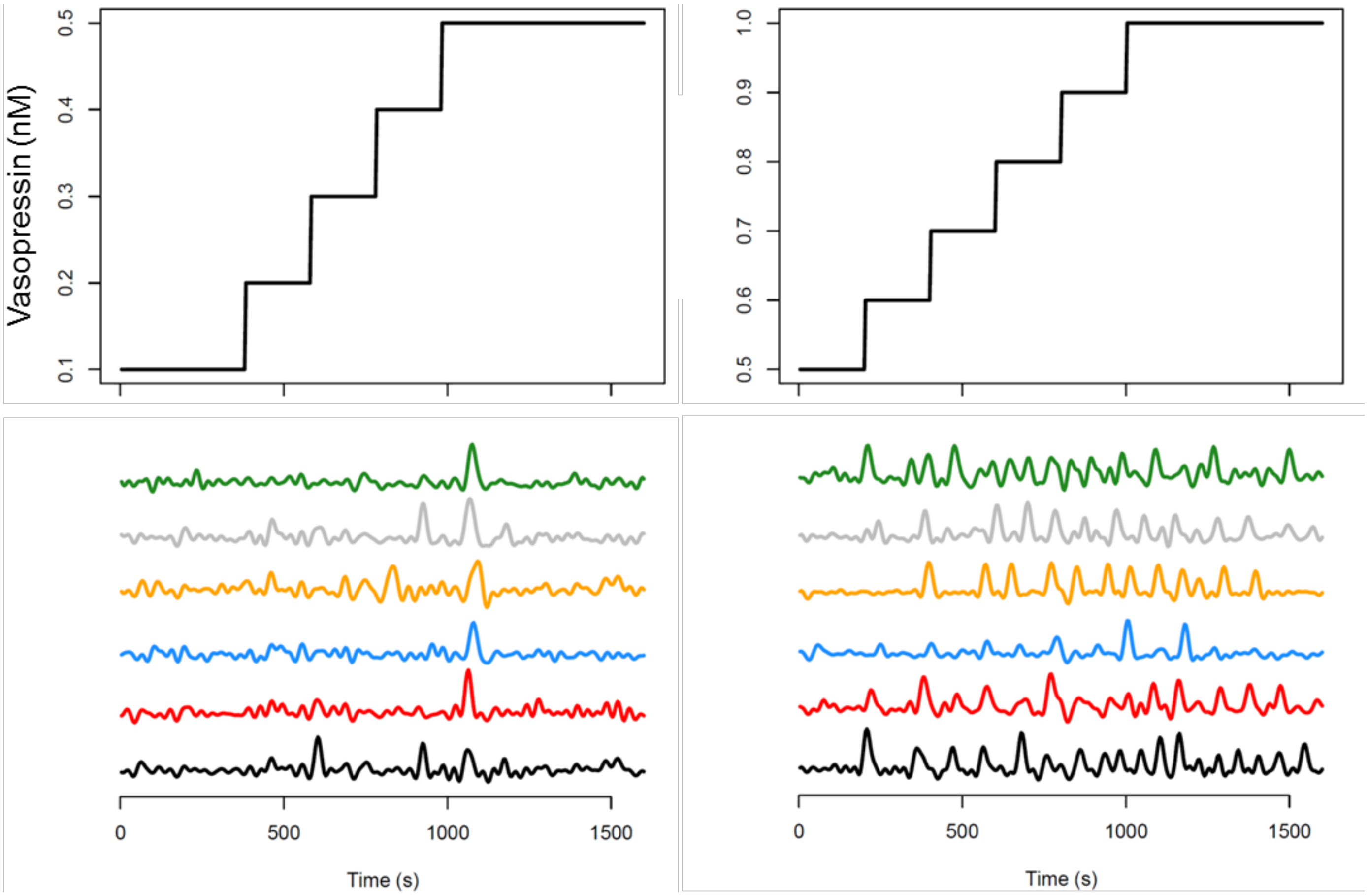

Figure S5: Hepatocytes in intact mouse livers exhibited high cytosolic Ca^{2+} spiking activity only at vasopressin stimulus concentrations higher than 0.5 nM. Note that even at high stimulus, some hepatocytes show cytosolic Ca^{2+} spikes at low frequencies (blue trace on the right)
