# Supplementary material for: Causality Analysis and Cell Network Modeling of Spatial Calcium Signaling Patterns in Liver Lobules
Source: Front Physiol. 2018 Oct 4;9:1377. doi: 10.3389/fphys.2018.01377 (PMC6180170; doi:10.3389/fphys.2018.01377)
Supplement: Supplementary file 7 [file Image_6.PDF]

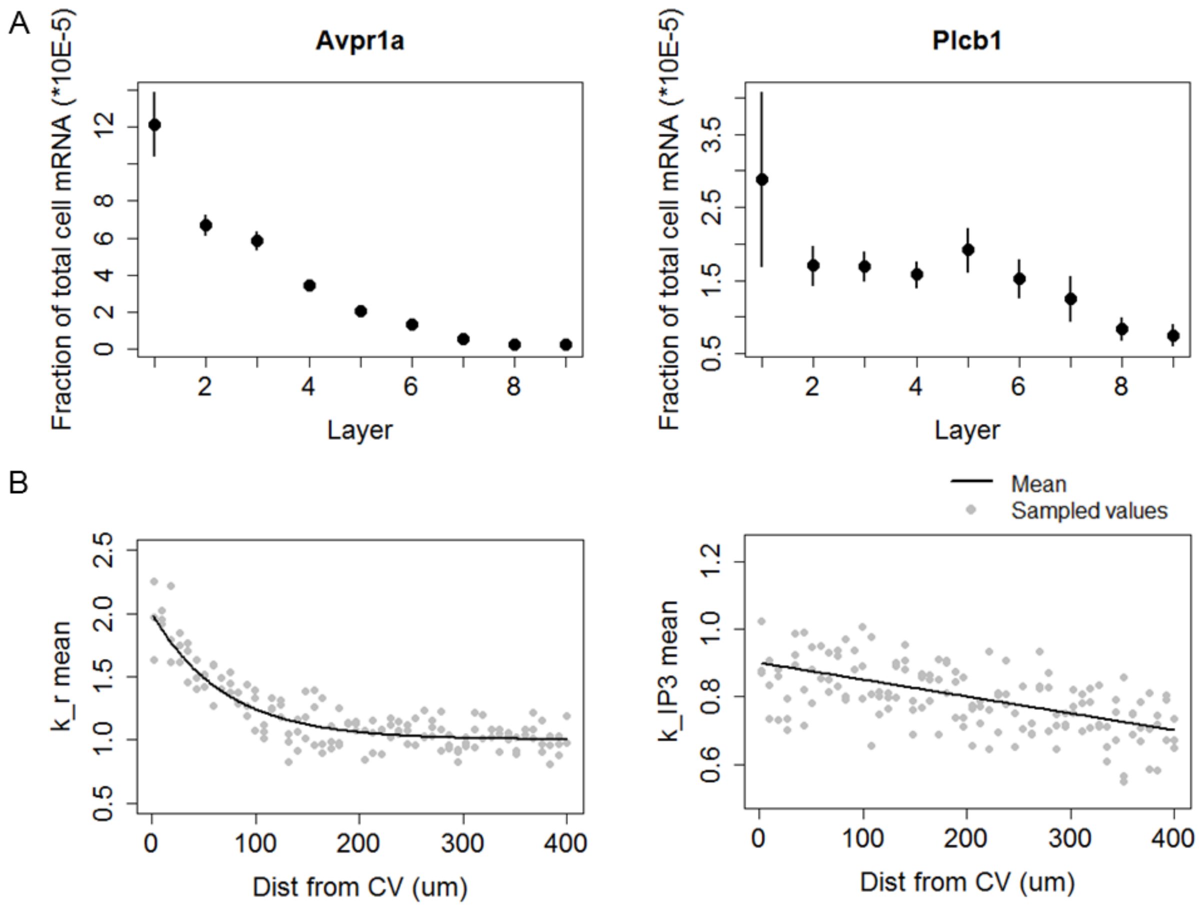

Figure S6: Initialization of parameter gradients using RNA-seq data in Halpern et al. 2017. The spatial gene expression profiles along the lobular porto-central axis for Avpr1a and Plcb1 are shown in (A) (data from Halpern et al. 2017). Parameters k_r and k_{IP3} in our model are proxies for Avpr1a expression and Plcb1 expression respectively. We approximated Avpr1a expression as an exponential function and Plcb1 as a linear function decreasing from the pericentral region (Layer 1) to the periportal region (Layer 9) within nominal parameter ranges. For a cell residing at a given distance from the central vein (CV), cell-intrinsic parameters were randomly drawn from truncated normal distributions with a mean value corresponding to the approximated functional form and standard deviations of 10% the local mean, such that parameter values were non-negative. The mean expression at a distance *d* from the CV was approximated by the following functions:

$$Avpr1a(d) = 1 + \exp(-d/70)$$

$$Plcb1(d) = 0.7 + 0.2*(1 - d/D);$$ *where* D = maximum distance of a cell from the CV

Mean values as a function of distance and 150 randomly sampled parameter values are shown in (B).
