# Supplementary material for: Causality Analysis and Cell Network Modeling of Spatial Calcium Signaling Patterns in Liver Lobules
Source: Front Physiol. 2018 Oct 4;9:1377. doi: 10.3389/fphys.2018.01377 (PMC6180170; doi:10.3389/fphys.2018.01377)
Supplement: Supplementary file 8 [file Image_7.PDF]

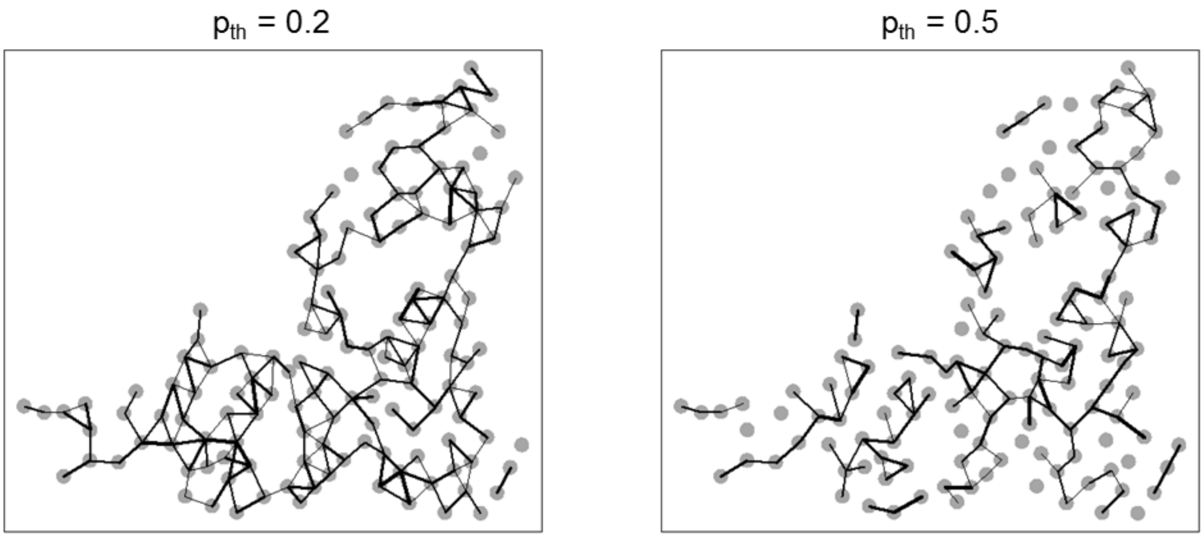

Figure S7: Mapping of G_{ij} values between hepatocytes in a cluster. Line widths are proportional to the G_{ij} value, with thicker lines representing higher G_{ij}.
