# Supplementary material for: Causality Analysis and Cell Network Modeling of Spatial Calcium Signaling Patterns in Liver Lobules
Source: Front Physiol. 2018 Oct 4;9:1377. doi: 10.3389/fphys.2018.01377 (PMC6180170; doi:10.3389/fphys.2018.01377)
Supplement: Supplementary file 9 [file Image_8.PDF]

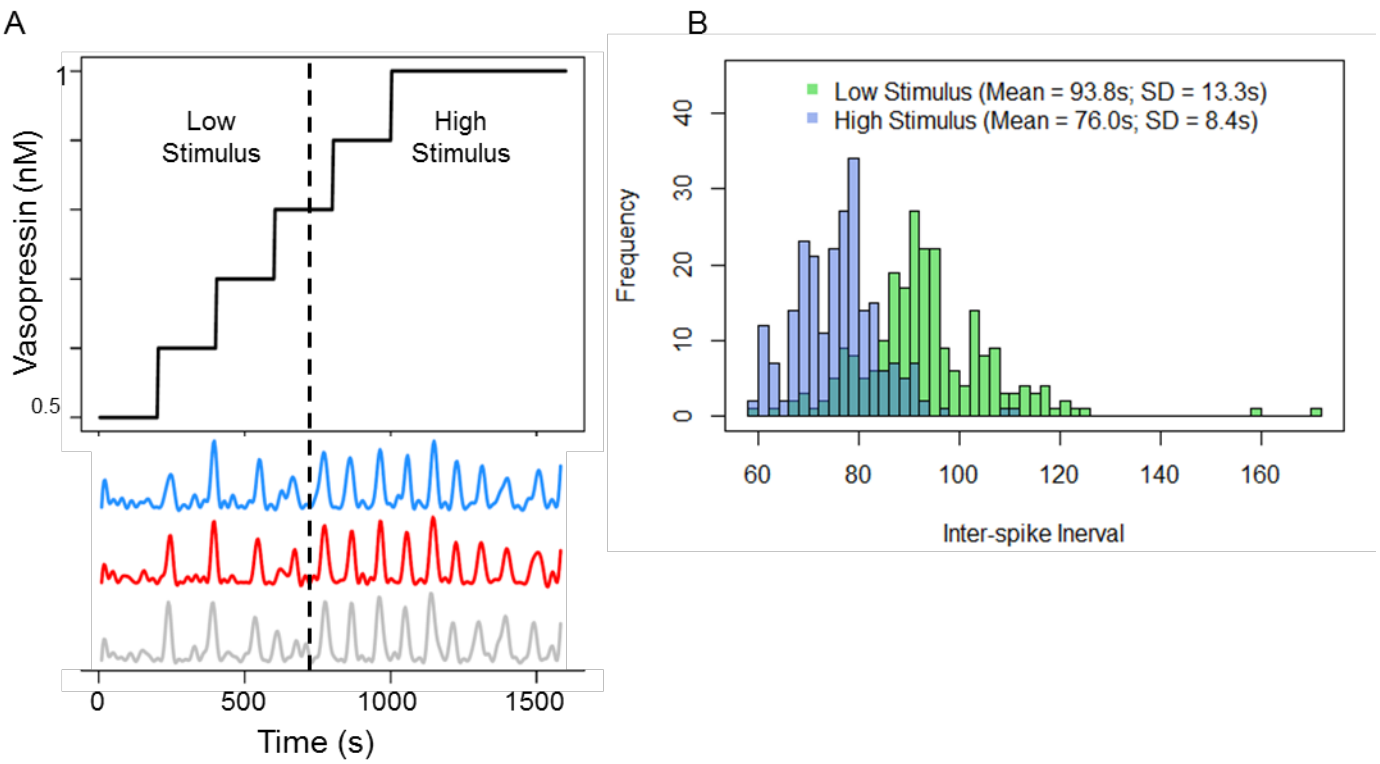

Figure S8: A: Division of the stimulus profile into low and high stimulus regimes. Cytosolic Ca^{2+} spike frequencies increases in hepatocytes in the high stimulus regime (Lower panel). B: Decrease in Cytosolic Ca^{2+} inter-spike interval (increase in spike frequency) at low and high stimulus regimes across 284 select hepatocytes.
