# Supplementary material for: Causality Analysis and Cell Network Modeling of Spatial Calcium Signaling Patterns in Liver Lobules
Source: Front Physiol. 2018 Oct 4;9:1377. doi: 10.3389/fphys.2018.01377 (PMC6180170; doi:10.3389/fphys.2018.01377)
Supplement: Supplementary file 10 [file Image_9.PDF]

Figure S9: Correlation network comparisons at low and high stimulus (continued)

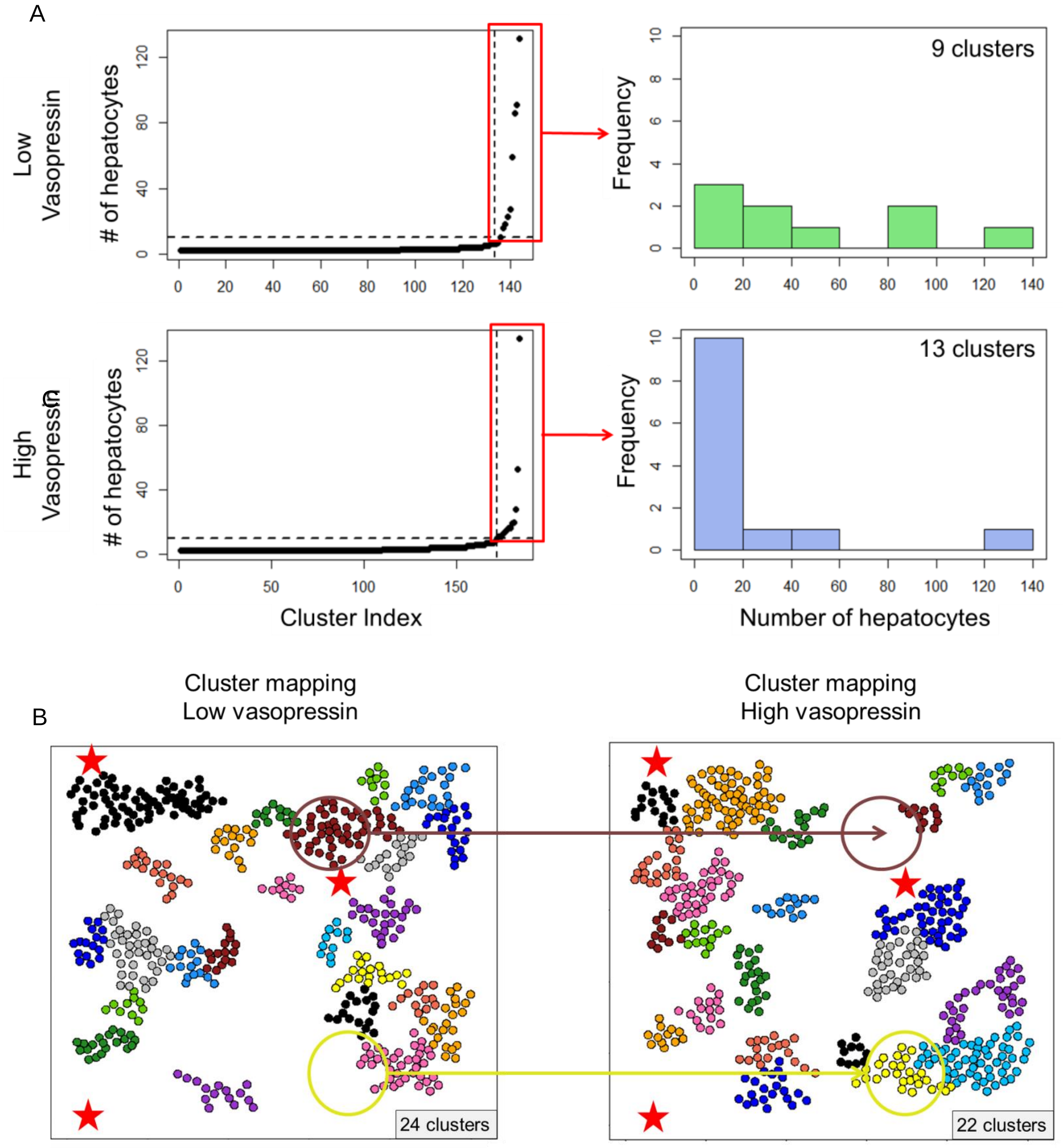

A: Correlation networks cluster sizes (left), cluster size distributions for large clusters (n >= 8, right) at low and high stimulus. B: Large clusters for low and high stimulus mapped on to the optical slice.
