# Supplementary material for: Causality Analysis and Cell Network Modeling of Spatial Calcium Signaling Patterns in Liver Lobules
Source: Front Physiol. 2018 Oct 4;9:1377. doi: 10.3389/fphys.2018.01377 (PMC6180170; doi:10.3389/fphys.2018.01377)
Supplement: Supplementary file 12 [file Image_11.pdf]

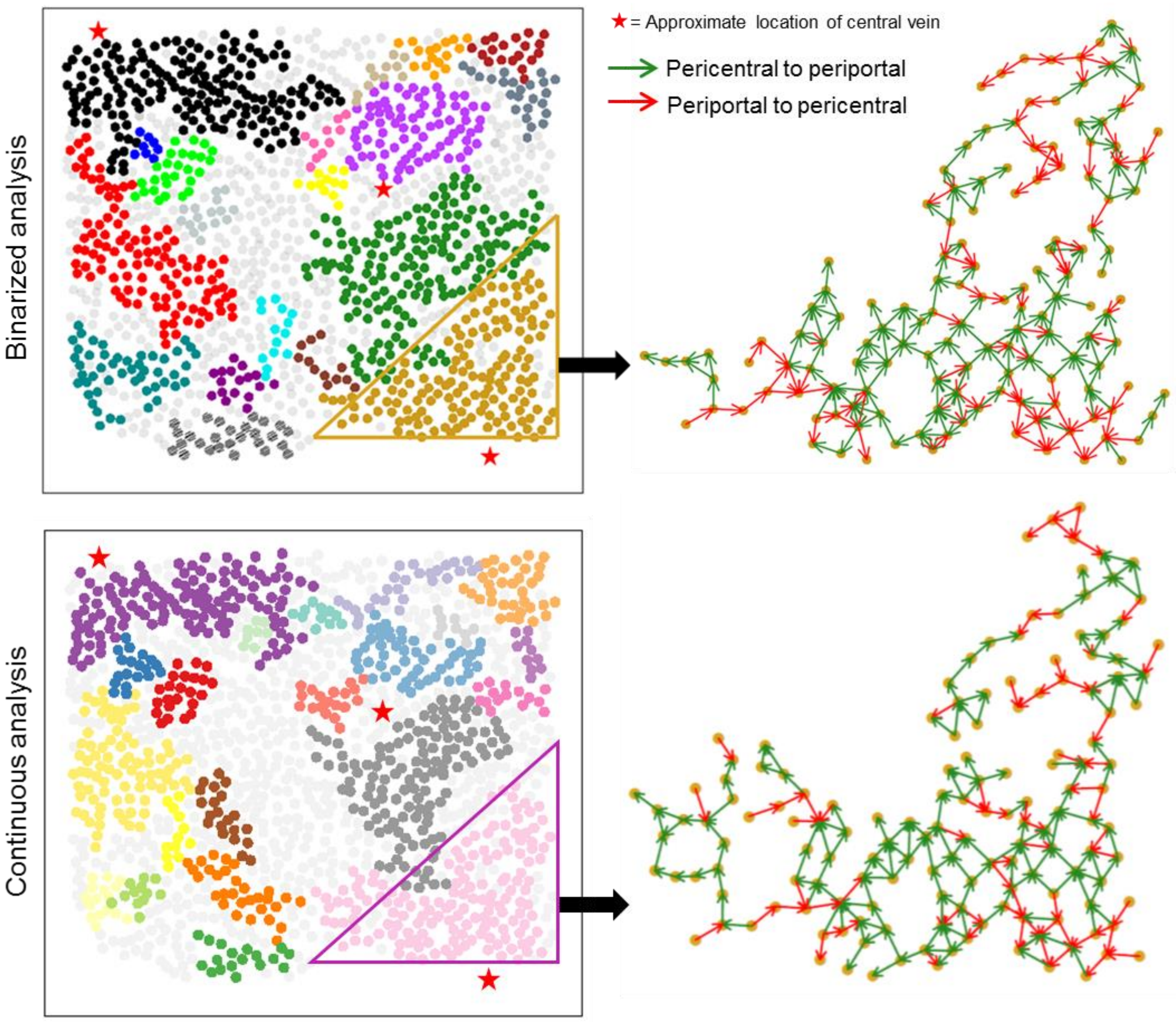

Figure S11: Comparison of TE network identification method used in this work (binarized analysis) and a JIDT (Lizier, 2014) implementation of Kraskov TE estimator using continuous cytosolic Ca^{2+} time series. In both cases the history value was set to 1, equivalent to a temporal difference of 4 seconds. The pre-processed cytosolic Ca^{2+} time series were used in both cases (Figure S1). Significant causal influences were identified as described in Methods Section 2.3.2. The cluster sizes, locations, and the causal edges between hepatocyte pairs are in close agreement in the two cases.
