# Supplementary material for: Causality Analysis and Cell Network Modeling of Spatial Calcium Signaling Patterns in Liver Lobules
Source: Front Physiol. 2018 Oct 4;9:1377. doi: 10.3389/fphys.2018.01377 (PMC6180170; doi:10.3389/fphys.2018.01377)
Supplement: Supplementary file 13 [file Image_12.pdf]

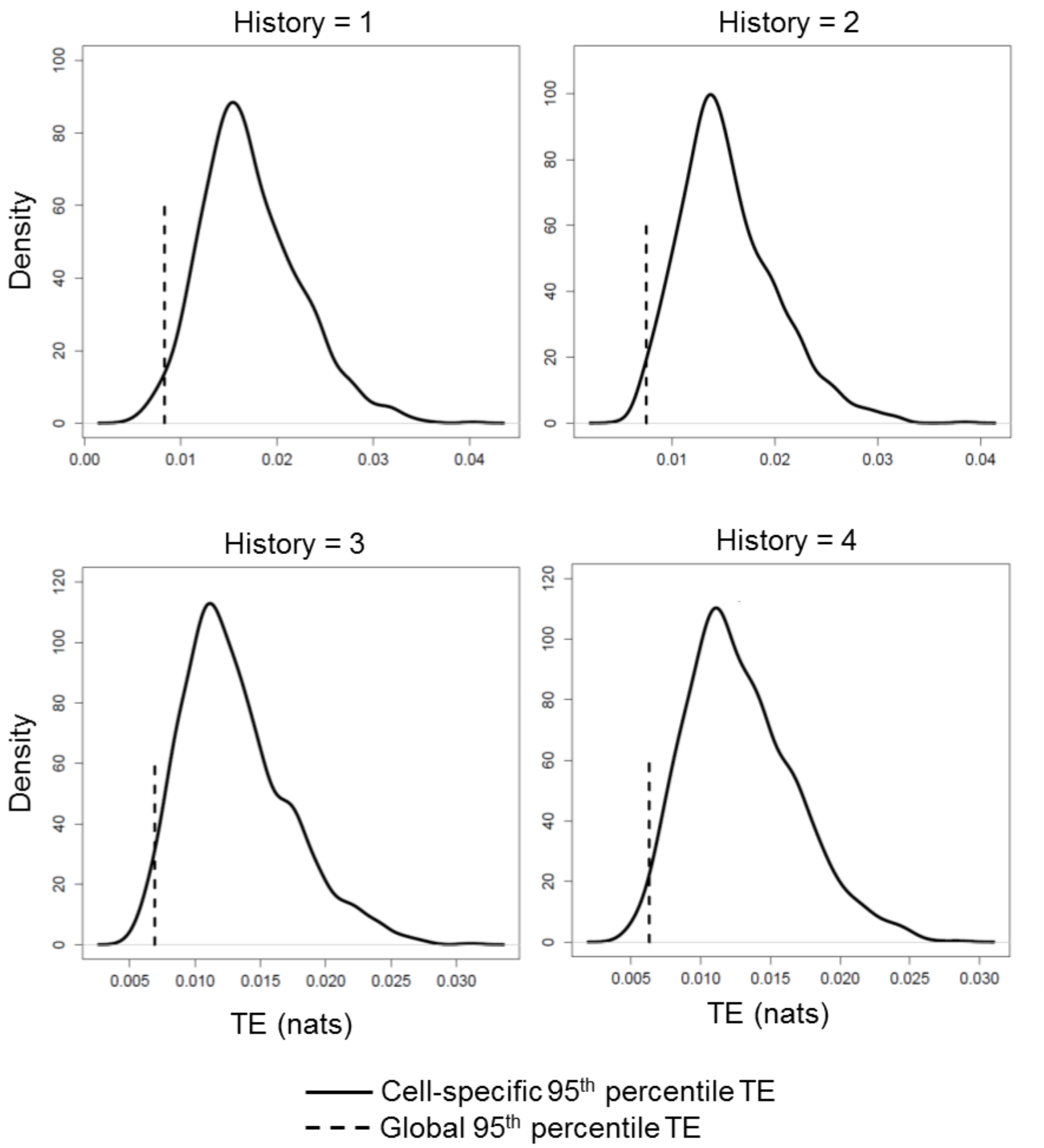

Figure S12: Using a global threshold in favor of cell-specific 95th percentile intensity in determining significant influence edges could lead to higher rates of false positives. A global TE threshold was estimated using the 95th percentile TE value for all pairwise TE estimates after scrambling the data for history values 1 to 4 (time lags of 4 to 16 seconds). The global threshold TE values are shown in the plots as dashed vertical lines. The solid lines show a distribution of cell-specific 95th percentile TE values for all 1300 hepatocytes without scrambling. The global TE threshold from scrambled data consistently lies at the lower end of the cell-specific TE threshold distribution for all values of the history. All pairwise TE values in both cases were estimated using the Kraskov estimator implemented in JIDT (Lizier et al. 2014).
